# Phase Morphology Dependence of Ionic Conductivity and Oxidative Stability in Fluorinated Ether Solid-State Electrolytes

**DOI:** 10.1021/acs.chemmater.4c00199

**Published:** 2024-05-09

**Authors:** Emily S. Doyle, Priyadarshini Mirmira, Peiyuan Ma, Minh Canh Vu, Trinity Hixson-Wells, Ritesh Kumar, Chibueze V. Amanchukwu

**Affiliations:** Pritzker School of Molecular Engineering, University of Chicago, Chicago, Illinois 60637, United States

## Abstract

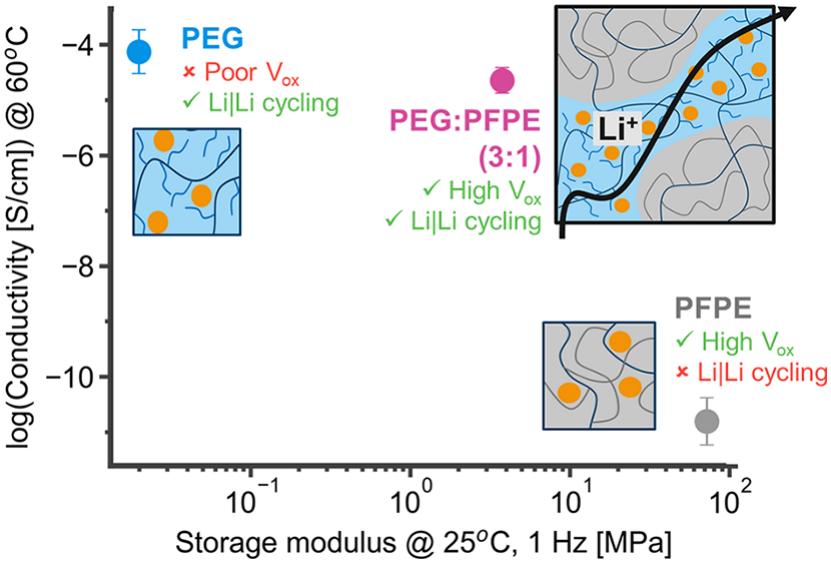

Solid-state polymer electrolytes can enable the safe operation of high energy density lithium metal batteries; unfortunately, they have low ionic conductivity and poor redox stability at electrode interfaces. Fluorinated ether polymer electrolytes are a promising approach because the ether units can solvate and conduct ions, while the fluorinated moieties can increase oxidative stability. However, current perfluoropolyether (PFPE) electrolytes exhibit deficient lithium-ion coordination and ion transport. Here, we incorporate cross-linked poly(ethylene glycol) (PEG) units within the PFPE matrix and increase the polymer blend electrolyte conductivity by 6 orders of magnitude as compared to pure PFPE at 60 °C from 1.55 × 10^–11^ to 2.26 × 10^–5^ S/cm. Blending varying ratios of PEG and PFPE induces microscale phase separation, and we show the impact of morphology on ion solvation and dynamics in the electrolyte. Spectroscopy and simulations show weak ion–PFPE interactions, which promote salt phase segregation into—and ion transport within—the PEG domain. These polymer electrolytes show promise for use in high-voltage lithium metal batteries with improved Li|Li cycling due to enhanced mechanical properties and high-voltage stability beyond 6 V versus Li/Li^+^. Our work provides insights into transport and stability in fluorinated polymer electrolytes for next-generation batteries.

## Introduction

Global electricity demand is expected to triple in the next 30 years with the mass commercialization of electrified vehicles, but the market lacks safe, high energy density batteries to support these applications.^1^ The switch from graphite to a lithium metal anode facilitates an order of magnitude increase in specific capacity from 372 mAh/g with graphite to 3860 mAh/g with lithium metal.^2−4^ Lithium metal anodes paired with high-voltage cathodes like lithium nickel manganese cobalt oxide (LiNi_*x*_Mn_*y*_Co_*z*_O_2_, *x* + *y* + *z* = 1, NMC), lithium cobalt phosphate (LiCoPO_4_, LCP), and lithium nickel manganese oxide (LiNi_0.5_Mn_1.5_O_4_, NMO) deliver promising energy densities, but most electrolytes do not have the necessary chemical and electrochemical stability to operate safely with these advanced electrodes.^5^ Safe electrolyte candidates must be developed to enable the implementation of high-voltage lithium metal batteries for a growing energy economy.

Solid-state polymer electrolytes can be a safe, high-performing electrolyte candidate as they eliminate flammability and leakage concerns, can suppress dendrite growth via cross-link density optimization, possess ideal mechanical properties to alleviate interfacial delamination during cycling, and are cost-effective and highly processable.^6−8^ However, the design process is currently hindered by the difficulty in optimizing both the ionic conductivity and oxidative stability simultaneously. The state-of-the-art poly(ethylene oxide) (PEO) electrolytes exhibit high conductivity on the order of 10^–3^ S/cm at 85 °C and 10^–5^ S/cm at room temperature but have low oxidative stability.^9^ Newer polymer designs with oxidatively stable functional groups such as nitriles, cyclic carbonates, and fluorines can achieve high oxidative stability but suffer from both instability at the lithium metal anode and low ionic conductivity.^10^ New polymer design strategies are needed to address this dual optimization challenge.

Fluorinated ether electrolytes have been widely studied in the liquid electrolyte literature, as fluorinated molecules are known to possess high oxidative stability, low flammability, and form ideal LiF containing degradation layers at both lithium metal and cathode interfaces.^7,11−15^ The literature iterates through various fluorinated ether electrolyte design strategies with varying fluorine density and molecular architecture.^4,16−19^ The degree of fluorine incorporation directly correlates with oxidative stability but often leads to lower ionic conductivity at a high fluorine content. Zhang et al. found that higher fluorine density decreases the ability of the molecule to coordinate with lithium ions.^4^ Previous work by our group has found that both the oxidative stability and conductivity of the fluorinated ether molecules can be fine-tuned by adjusting the solvation structure for optimal ether oxygen (EO)–Li^+^ interaction.^18^ The ability to fine-tune the fluorinated ether molecular structure to achieve high ionic conductivities and high oxidative stability simultaneously makes this functional class ideal for polymer electrolyte design, which struggles to meet these targets.

While the liquid fluorinated ether electrolytes have been optimized to achieve enhanced conductivity and oxidative stability,^11,12,20^ in practice, the solid counterparts still suffer from low conductivity that hinders their realistic implementation in battery cells.^16,17^ Previously, cross-linked perfluoropolyether (PFPE) electrolytes have been studied, and they show poor ionic conductivity. Grey et al. investigated a cross-linked PFPE electrolyte using solid-state magic angle spinning nuclear magnetic resonance (MAS NMR) spectroscopy to understand the solvation and mobility of Li ions in the polymer electrolyte.^22^ They observed that low conductivity stems from the poor coordination of Li^+^ with the PFPE backbone that forces nonlabile Li^+^-TFSI^–^ coordination, resulting in low Li^+^ mobility in the polymer. Balsara et al. created two solid PFPE systems by cross-linking pure PFPE methacrylate components in one variant and by cross-linking the methacrylate PFPE with acrylate-functionalized siloxane cross-linkers in another.^21^ The presence of the siloxane cross-linker was found to increase the conductivity of the polymer over the pure, solid PFPE by 2 orders of magnitude due to the preferential interaction of salt with the siloxane units. At high salt loadings, this trend broke down, and the salt interaction with PFPE outweighed solvation by the siloxane, diminishing the positive impacts of the siloxane moiety on the conductivity. Insights from these studies demonstrate the correlation between poor conductivity and poor ion solvation caused by the weak distribution of electron density along the PFPE backbone. The CF_2_ groups along the backbone pull electron density from the EO atoms, interrupting the ideal helical ether chelation structure formed between EO atoms and lithium ions in glyme and PEO electrolytes.^12,23−25^ Therefore, a successful fluorinated ether polymer electrolyte must increase access to electron-dense functional groups to increase the overall ionic conductivity beyond that obtained in conventional PFPE systems.

The addition of PEO to the PFPE matrix is expected to introduce conductivity to the electrolyte by adding EO units that can solvate and transport lithium ions. Past work by the Balsara and DeSimone groups have extensively studied liquid PFPE electrolytes.^11,12,26^ The addition of short ether-containing end groups to liquid PFPE led to microphase separation, as observed via small-angle X-ray scattering.^26^ The addition of short PEO homopolymer chains, termed poly(ethylene glycol) (PEG), in the liquid ternary mixture of PEG–PFPE–lithium bis(trifluoromethanesulfonyl)imide (LiTFSI) was also investigated, which revealed that miscibility of the three compounds spans only a small compositional window, with the salt causing exacerbated phase separation as its content increases.^20^ This phase immiscibility extends into the solid PEG–PFPE cross-linked system, where the increasing length of PEG and PFPE components led to larger length-scale phase separation.^27^ In these systems, increasing the PFPE content led to smaller, tortuous PEG domains with agglomerated PFPE phases.

Phase separation in electrolytes tends to yield an ideal combination of the individual component properties. This is leveraged in block copolymers and ceramic hybrids where one phase contributes ionic conductivity and another contributes mechanical stability.^28−30^ The miscibility of the solid ternary mixture of PEG–PFPE–LiTFSI has not been reported, but salt addition is expected to affect the miscibility of the PEG and PFPE chains.^31^ Furthermore, the exact mechanism of the salt phase behavior and solvation is unknown in these two-phase systems and is desired to understand the role that fluorinated components play in stabilizing the polymer material.

**Figure 1:**
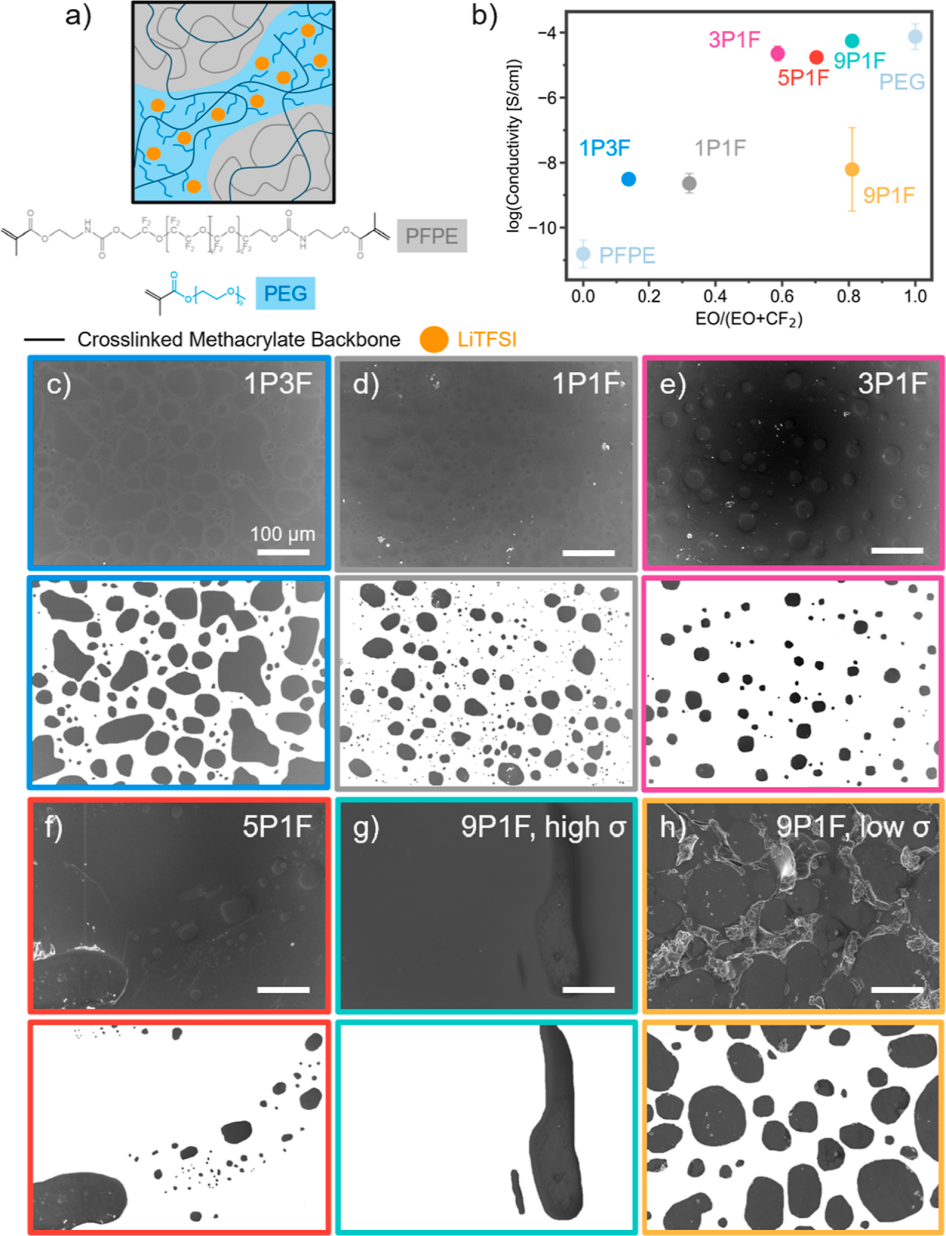
Effects of polymer design on system properties. (a) Schematic diagram showing the architecture and phase morphology of the PEG/PFPE (*x*/*y*) (*x*P*y*F) polymer electrolyte systems. (b) Ionic conductivity trends at 60 °C with *r* = 0.05 (Li^+^/EO) LiTFSI as the PEG/PFPE ratio varies. Scanning electron microscopy (SEM) and correlating mask images (PEG phase = white and PFPE phase = dark) of the cross-linked polymers for (c) 1P3F, (d) 1P1F, (e) 3P1F, (f) 5P1F, (g) 9P1F high-conductivity sample, and (h) 9P1F low-conductivity sample electrolytes with *r* = 0.05 LiTFSI. Scarring in the 9P1F low-conductivity sample is due to mechanical aggravation during sample preparation, as the 9P1F samples were extracted from coin cells to study the correlation between the morphology and conductivity. Scale bar = 100 μm. *x*P*y*F, where *x*/*y* is the molar ratio of PEG chains to PFPE chains.

Herein, we designed a polymer blend system that achieves increased ionic conductivity while maintaining the oxidative stability of PFPE via the inclusion of PEG for optimal ion solvation and mobility. We cross-link a PEG methyl ether methacrylate compound with nine dangling EO units to the PFPE to create percolated ion transport pathways through the oxidatively stable PFPE network. Figure 1a shows a schematic of the final cross-linked network with phase separation induced by the hydrophobicity/philicity of the PFPE and PEG components, respectively. Using a suite of thermal, modeling, and spectroscopic tools, we probed the effects of PEG versus PFPE contributions to ion complexation and transport. We demonstrate 6 orders of magnitude increase in conductivity at 60 °C from pure PFPE to the optimized polymer blend and show that despite geographically limited PFPE presence on the polymer|electrode interfaces, the fluorinated moiety introduces >1 V increase in oxidative stability over the pure PEG polymer variant. Insights for enhanced ion solvation and mobility obtained from this work will inform the synthesis and molecular engineering of future solid-state fluorinated ether electrolytes.

## Results and Discussion

### Copolymer Phase Separation

The PEG–PFPE homopolymer mixture was cross-linked in the presence of LiTFSI via the methacrylate polymer end groups to form a continuous network. Five blends with this architecture were studied and are referenced as *x*P*y*F, where *x*/*y* is the molar ratio of PEG chains to PFPE chains. Miscibility and salt–polymer interactions have not been investigated in the solid-state PEG–PFPE–LiTFSI ternary system, and the salt is expected to constrict the polymer miscibility window and create more drastically phase-separated morphologies, as compared to the PEG–PFPE mixture.^27^Figure S1 shows that salt addition appears to extend phase separation between the PFPE and PEG phases. As the EO content in the cross-linked PEG–PFPE increases, ionic conductivity increases significantly, leading to films that approach the conductivity of pure cross-linked PEG and have 6 orders of magnitude higher ionic conductivity compared to pure PFPE (Figures 1b and S2). These electrolytes contain *r* = 0.05 LiTFSI, where *r* quantifies the ratio between Li^+^ and EO units. Due to the phase separation and low complexation between fluorine-shielded EO units on the PFPE chain and Li^+^, only EO units on the PEG chain are counted in this ratio. Detailed calculations are provided in the Supporting Information, and a brief study of the salt content effect on ionic conductivity is provided in Figure S3.

The morphologies obtained by altering the PEG/PFPE ratio are shown in Figure 1c–h, and there is a strong correlation between the morphology and the trend in conductivity shown in Figure 1b. Based on energy-dispersive X-ray (EDX) spectroscopy analysis of the two phases (Figure S4), PFPE is engulfed in a PEG matrix, which becomes narrower and more tortuous as the PFPE content increases. The optimal conductivity occurs when the mixture contains three PEG chains per PFPE chain or PEG/PFPE (3:1), which we term 3P1F, pictured in Figure 1e. The 3P1F ionic conductivity is highly reproducible, despite slight variations in morphology between batches (Figure S5). The morphology dependence of the conductivity is highlighted in the 9P1F samples, where the phase separation is not homogeneous across the polymer surface, and some samples have more PFPE phase surface area than others, creating a divide in the conductivity data. The higher conductivity regime sample has PEG as the primary phase across the sample surface area, and the low conductivity regime has a higher PFPE surface area, as detailed in the 9P1F SEM images in Figure 1g,h.

### Ion Solvation in Dual Phase System

**Figure 2:**
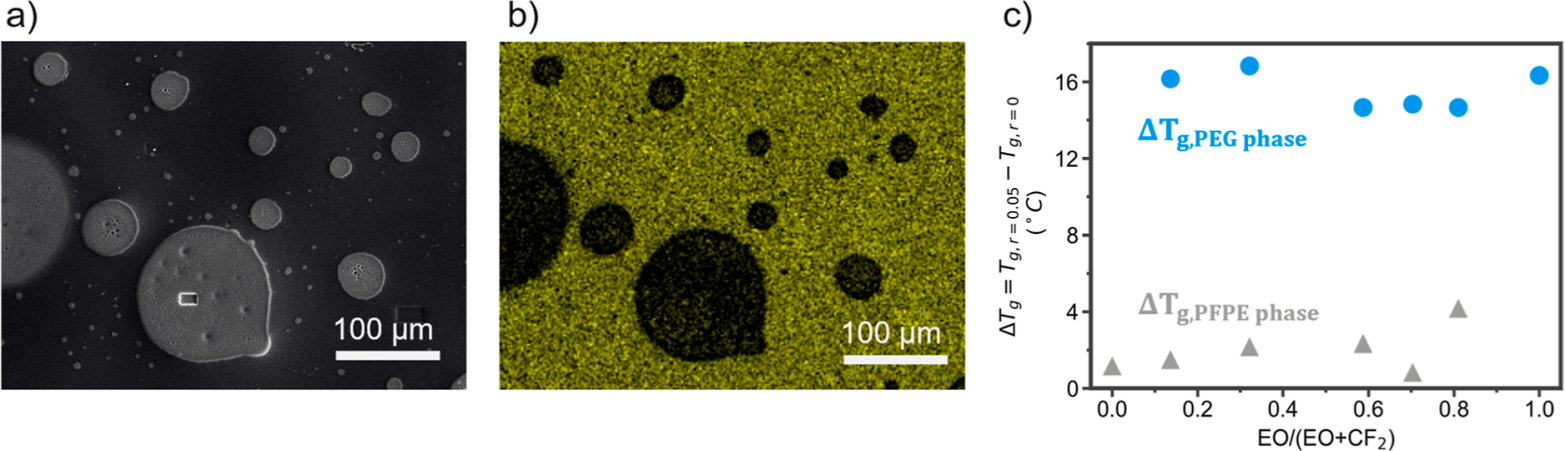
Phase separation. (a) SEM image and (b) sulfur Kα1 SEM EDX mapping of the 3P1F film. Sulfur originates from the TFSI anion, with the SEM EDX mapping of sulfur showing the distribution of TFSI anions in the PEG phase of the film. A rectangular burn mark about 10 μm in length is observed in the largest PFPE phase of (a) from this measurement. (c) Differential scanning calorimetry (DSC) analysis showing the difference in *T*_g_ for both the PEG and PFPE phases between *r* = 0 and *r* = 0.05 LiTFSI samples.

It is apparent that phase separation plays an important role in modulating the conductivity, but detailed studies of ion–ion interactions, ion–polymer interactions, and transport mechanisms are needed to fully understand the impact of this phase separation. The observed phase separation is induced by hydrophobic–hydrophilic repulsion of PFPE and PEG chains.^27,32^ The LiTFSI salt will preferentially coordinate with the more hydrophilic PEG which solvates the ion and facilitates favorable binding interactions. EDX paired with SEM was used to probe this phenomenon. By mapping the sulfur distribution on the surface of the PEG/PFPE (3:1) (3P1F) sample, it is clear in Figure 2a,b that LiTFSI is preferentially located in the PEG phase, while the PFPE domains have only dilute quantities of the TFSI anion present.

Differential scanning calorimetry (DSC) gives a quantitative analysis of the phase separation of LiTFSI into the PEG phase. Phase-separated polymers exhibit glass-phase transition temperatures (*T*_g_) characteristic of each phase.^20,33^ In the *x*P*y*F systems, the materials show a first *T*_g_ near −110 °C and a second *T*_g_ near −40 °C, indicative of the PFPE and PEG phases, respectively (Figure S6).^27^Figure 2c shows the difference in both the PEG phase *T*_g_ (Δ*T*_g,PEG phase_) and the PFPE phase *T*_g_ (Δ*T*_g,PFPE phase_) for each polymer ratio resulting from the addition of salt into the polymer. The difference between the pristine (*r* = 0) and the samples with LiTFSI (*r* = 0.05) for pure PFPE and pure PEG is shown at the 0 and 1 ends of the *x*-axis, respectively. The difference in *T*_g_ for the PEG phase aligns evenly around 16 °C for the PEG phase and around 2 °C for the PFPE phase, regardless of PEG/PFPE ratio. The large increase in PEG phase *T*_g_ indicates that LiTFSI interacts with the PEG backbone, creating physical cross-links that limit the mobility of the PEG phase. This shows that LiTFSI strongly favors interaction with PEG and will phase-separate into the PEG matrix even at low PEG concentrations. The 9P1F sample exhibits a 4 °C increase in the PFPE phase *T*_g_ with the addition of salt, which may stem from the increased interaction of PFPE and PEG chains in the high PEG content film. The PFPE phase *T*_g_ is nearly unobservable in Figure S6e, showing that the PFPE phase is affected by the high PEG content.

The ion solvation environments were ascertained by using spectroscopic techniques. Raman was used to determine the degree of salt dissociation, while NMR was used to provide insights into the solvation environments of both the Li^+^ and TFSI^–^ ions. Raman spectroscopy probes the degree of salt dissociation via the stretching mode of the S–N–S bond of TFSI^–^. This vibrational mode shifts depending on the coordination state of the ion. Free TFSI^–^ resonates near 740 cm^–1^, and a blue shift to 744–748 cm^–1^ is observed for anions interacting with Li^+^.^25,34^ The degree of LiTFSI association quantifies how labile Li^+^ is in each polymer environment to develop intuition for the polymer backbone’s interaction with the ions.

**Figure 3:**
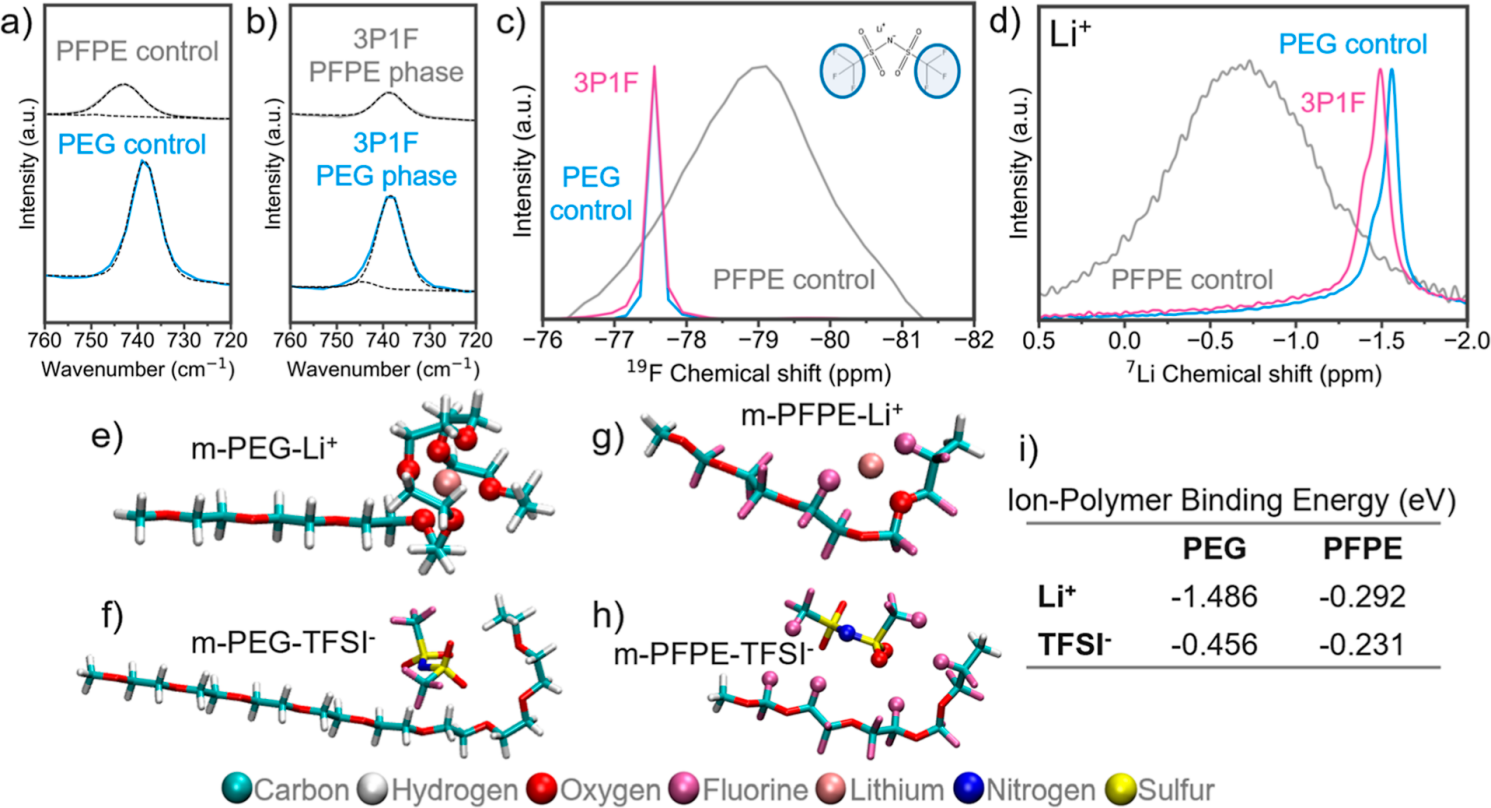
Salt dissociation and polymer–salt solvation. Raman analysis of the salt dissociation via the shifting of the S–N–S stretching mode at ∼740 cm^–1^ for (a) control PFPE and PEG samples and (b) PFPE and PEG phases in the 3P1F sample. (c) Normalized ^19^F MAS NMR spectra of the PEG and PFPE control electrolytes and the 3P1F electrolyte in the TFSI^–^ fluorine signal domain. (d) Normalized ^7^Li MAS NMR spectra of the lithium ion in the PEG and PFPE control electrolytes and the 3P1F electrolyte. Density functional theory (DFT)-optimized solvation structures for the (e) PEG macromonomer (m-PEG)-Li^+^, (f) m-PEG-TFSI^–^, (g) PFPE macromonomer (m-PFPE)-Li^+^, and (h) m-PFPE-TFSI^–^ complexes. Each atom is represented by the following colors: cyan (C), white (H), red (O), mauve (F), pink (Li), blue (N), and yellow (S). The atoms represented by balls are <3.25 Å from another atom on the other molecule, while all other atoms are represented by their colors at the angles of the 3D line diagram. (i) Binding energies (in eV) of Li^+^ and TFSI^–^ to the PEG and the PFPE backbones, as calculated from the relaxed structures in (e–h).

Pure homopolymer networks of PFPE and PEG with a Li^+^/EO ratio of 0.05 (*r* = 0.05) were tested to determine the ability of each component to solvate LiTFSI. These control spectra are shown in Figure 3a. The PFPE sample demonstrates contact ion pairs with a primary peak at 743 cm^–1^ and ion aggregates evident in a minor peak at 749 cm^–1^ that accounts for 1.12% of the total peak area. This highlights PFPE’s low solvation strength due to the withdrawal of electron density by the CF_2_ units away from the EO sites that typically chelate Li^+^. In contrast, the PEG sample fully solvates LiTFSI, as evidenced by the single peak at 738 cm^–1^.

Similarly, the 3P1F *r* = 0.05 sample shows ion dissociation with peaks at 738 cm^–1^ in both the PFPE and the PEG phases. These spectra were taken from the same polymer sample, with irradiation preferentially conducted on the individual phases. The spot resolution of the instrument is 0.6 μm, allowing such discernment of phases. The EDX results affirmed that the LiTFSI phase separates into the PEG phase; therefore, the TFSI^–^ signal in the PFPE phase is indicative of a dilute amount of salt. Figure 3b shows the lower intensity of the salt peak in the PFPE versus the PEG phase, where the ratio of the PFPE phase peak to the PEG phase peak is 0.56. The full spectra normalized to the ester peak from the methacrylate end groups are shown in Figure S7 and clearly delineate the differences in the LiTFSI peak intensity. The dissociation of ions in the PFPE phase is likely due to the interaction with PEG chains that became entrapped in the PFPE domain during the fabrication process. A minor peak designating contact-ion pairs appears in the 3P1F PEG phase, which shows that *r* = 0.05 is the maximum amount of salt needed to achieve full dissociation in this system based on the known stoichiometry of EO–Li^+^ coordination.^35^ The dominance of the dissociation peak shows that there is minimal ion clustering, and ideal ion–polymer interactions exist in the PEG phase of the 3P1F sample. The same trends are seen in the remaining PEG/PFPE polymer ratios (Figure S8).

### Anion–Polymer Interactions

MAS NMR probes the electron density around the lithium ion and the fluorine atoms in TFSI^–^ to observe the solvation environments of the ions within the polymer matrix. The spectra for the Li nuclei and the F nuclei were taken in pure PEG and PFPE *r* = 0.05 LiTFSI samples to determine the standard ion solvation environment in each polymer system. Control samples for PEG and PFPE *r* = 0 were prepared, and the spectra in Figure S9 confirm that the lithium and fluorine peaks investigated herein are due to the addition of LiTFSI. The ^19^F MAS NMR is shown in Figure 3c, and the spectra of all polymer blends and LiTFSI along with analysis of the peak chemical shift values and line widths are presented in Figure S10. The TFSI^–^ peak in PFPE is broad in contrast to the sharp TFSI^–^ peak in PEG. This suggests that the salt has a lower mobility in the PFPE phase than in the PEG phase due to ion aggregation. In addition to its low mobility, the anion may exist in many solvation states, where no state has optimal preference, showing the poor solvation of the salt in the PFPE environment. The sharpness of the PEG peak shows the uniformity of solvation structure types.

The ^19^F chemical shift for TFSI^–^ in the PEG/PFPE (3:1) (3P1F) sample aligns with TFSI^–^ in the PEG control at −77.4 ppm. This alignment confirms that the salt phase separates in the composite sample into the PEG phase, as seen qualitatively in the EDX sulfur mapping in Figure 2b, where it assumes solvation states identical to those observed in the pure PEG system. The line widths of these two TFSI^–^ peaks are equivalent, showing that TFSI^–^ has mobility similar to TFSI^–^ in pure PEG, further confirming the phase separation. The ^19^F MAS NMR results indicate the absence of F–F interactions between the TFSI^–^ and the PFPE backbone, as the –CF_3_ F nuclei of the TFSI^–^ are in the same solvation environment in the pure PEG sample and in the polymer composite samples. Any interactions between the PFPE backbone and the anion in the 3P1F sample would induce an upfield chemical shift of the TFSI^–^ peak toward its more shielded position in the pure PFPE sample.^4^ The TFSI^–^^19^F chemical shift in the polymer blend aligns with the shift in the pure PEG in all of the PEG/PFPE ratios (Figure S10), and interestingly the line width of the peak increases with the PFPE content, possibly indicating the inhibition of PEG mobility and ion mobility as the PFPE fraction increases and constricts the PEG phase volume.

The CF_2_ peaks on the PFPE backbone can be used to probe the possible F–F interaction between the polymer and the TFSI^–^ anion further. A few studies have reported favorable F–F interactions between the solvent and salt that increase the lithium transference number.^12,21,36^ This interaction is visible in the shifting of the CF_2_ backbone peaks in the ranges of [−91, −88] and [−56, −51] ppm. Previous studies state that F–F interaction is present when these backbone peaks shift upon the addition of salt to the polymer system.^15,22^ In the pure PFPE case, these peaks do not shift between the *r* = 0 and 0.05 LiTFSI content polymers, as shown in Figure S11. Similarly, the 3P1F sample has no shifting in the CF_2_ backbone peak regions upon the addition of LiTFSI. It is intuitive that no shifting will occur in the composite polymer sample CF_2_ backbone signals since the LiTFSI primarily resides in the PEG phase and does not exist in PFPE solvation environments. However, it is surprising that the pure PFPE sample shows no backbone peak shifting with LiTFSI present within the fluorinated matrix based on the observations of F–F interaction in the fluorinated ether liquid and solid electrolyte literature.^12,21,36^ However, according to Pauling’s principle, fluorine atoms only show weak coordination due to their low polarizability.^4,32,37^ The dispersion of electron density between alpha-fluorine atoms in CF_2_ and CF_3_ groups further inhibits F–F interactions in the PFPE–TFSI^–^ pairing.^4,15,32^ Additionally, while PFPE–TFSI^–^ interactions have been seen in liquid systems, the cross-linked PFPE network likely restricts CF_2_ rearrangement and leads to nonideal orientations for F–F interactions, leading to poor PFPE-TFSI^–^ interactions in the solid PFPE phase.^22,32^ This absence of PFPE–TFSI^–^ interactions informs the formation of salt clusters demonstrated in the PFPE sample Raman spectra in Figure 3a.

### Cation–Polymer Interactions

**Figure 4:**
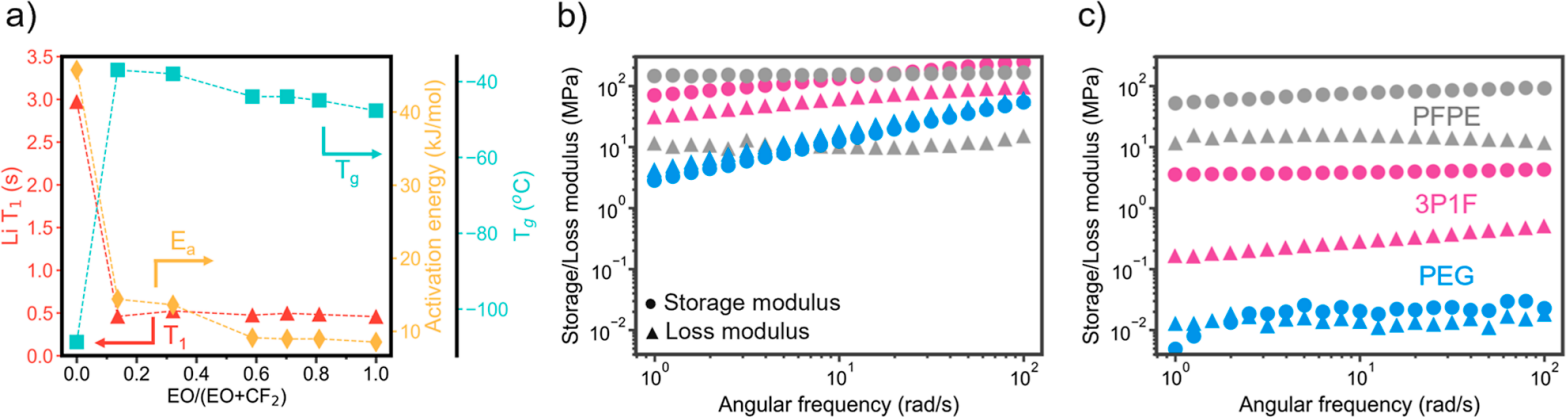
Ion and polymer mobility. (a) Transport dynamics parametrized by Li^+^ T_1_ from ^7^Li MAS NMR, VTF fit activation energy (*E*_a_), and PEG-phase glass-transition temperature (*T*_g_) in the *x*P*y*F films as the EO content increases. Notably, EO/(EO + CF_2_) = 0 is representative of the pure PFPE film, so *T*_g_ represents the PFPE *T*_g_. Dynamic mechanical analysis (DMA) results at (b) −50 °C and (c) 25 °C for pristine (*r* = 0) PFPE, 3P1F, and PEG samples.

^7^Li MAS NMR further demonstrates the phase separation of LiTFSI into the PEG phase with the Li^+^ signal in PEG/PFPE (3:1) (3P1F) within 0.07 ppm of the Li^+^ signal in the PEG control sample in Figure 3d. Helical EO arrangement in PEG provides a more electron-dense environment for Li^+^, yielding an upfield shift from LiTFSI in the PFPE sample, which explains the ease of continual ion hopping and dissociation between EO solvation sites in PEG.^38^ As the PFPE content increases, the Li^+^ peak shifts downfield (Figure S12). The shift in the Li^+^ environment in these samples could be due to the frustration of PEG rearrangement, restricting the formation of an ideal, electron-rich Li^+^–EO helical solvation structure. This idea is supported by the gradual increase in the PEG phase *T*_g_ (Figure 4a) as the PFPE content increases, which shows the heightened immobility of PEG as the phase is constricted. This will be discussed in more detail later.

### Computational Investigation of Ion Solvation Energetics

Binding energy calculations were conducted using density functional theory (DFT) to further inform the salt complexation mechanisms and intuition for phase separation. A previous study by the Forsyth group investigated the impacts of fluorinated backbones on anion and cation coordination in a PEG-block-PFPE, sodium bis(fluorosulfonyl)imide (NaFSI)-based system.^36^ They showed that the fluorinated components increased the anion’s coordination with the backbone to increase the lithium transference number. Here, we conducted a DFT study on cation-macromonomer and anion-macromonomer representative systems to understand the complexation of the anion and cation in each of the phase domains and explain the phase separation. The macromonomer systems were used as opposed to the full polymer to minimize computational time. The PEG macromonomer (m-PEG) has nine EO units, which is the same length as that of the PEG side chain used in the *x*P*y*F class of polymers. The PFPE macromonomer (m-PFPE) contains five EO units, which is half the length of PFPE used in the *x*P*y*F polymers. More details can be found in the Methods section. First, solvation structures were determined for each system, m-PEG-Li^+^, m-PFPE-Li^+^, m-PEG-TFSI^–^, and m-PFPE-TFSI^–^, by finding the relaxed conformations of the m-PEG and m-PFPE chain segments with the respective ions using Gaussian 16.^39^ These relaxed complexes are shown in Figure 3e–h. The structural relaxations were carried out using implicit solvation to account for the effects of the polymer dielectric environment. In this model system, the relative binding energies of Li^+^ and TFSI^–^ to the polymer backbones show their respective proclivities for coordination with the backbone atoms.

m-PEG-Li^+^, Figure 3e, shows the expected helical ether solvation structure with six EO groups coiling around the Li ion in excellent agreement with previous reports.^23−25^ m-PFPE-Li^+^, Figure 3g, shows very loose coordination in comparison to the m-PEG solvation environment. Whereas m-PEG encapsulates Li^+^ with an average EO-to-Li^+^ distance of 2.1 Å, the m-PFPE chain bends around the lithium ion with larger interatomic distances between the backbone atoms and Li^+^. The closest electron donor on m-PFPE is 3.1 Å from Li^+^, demonstrating the weak interaction between the m-PFPE chain and Li^+^, since the strong polymer–Li^+^ coordination typically exhibits a first solvation shell within ∼2–2.5 Å of the Li ion.^38,40,41^ Additionally, only three electron-dense atoms are within a 3.2 Å radius of Li^+^ in the m-PFPE system as compared to the m-PEG system where six EO atoms are less than 2.2 Å from Li^+^. This leads to higher coordination of Li^+^ by the polymer as compared to the anion in the m-PEG system and higher salt aggregation in the m-PFPE system as shown spectroscopically in Figure 3a.

The substitution of Li^+^ with TFSI^–^ in each system, shown in Figure 3f for m-PEG and Figure 3h for m-PFPE, reveals much weaker polymer–anion interactions, as demonstrated in previous polymer-TFSI^–^ coordination studies,^41^ with the closest interatomic distance between the anion and the polymer backbone >3 Å in both the m-PEG and m-PFPE systems. In the m-PEG-TFSI^–^ and m-PFPE-TFSI^–^ systems, sulfonyl oxygen atoms on TFSI^–^ are oriented toward the chain backbones, with the CF_3_ end groups of TFSI^–^ pointing away from the backbones. In the m-PFPE system, this optimal configuration highlights the low preference for F–F interactions between the anion and the polymer.

Binding energies calculated from these solvation structures are listed in Figure 3i. These values corroborate the tendency for phase separation of the salt into the PEG phase. The m-PEG–Li^+^ solvation has a significantly stronger binding energy than the m-PFPE–Li^+^ system by 1.19 eV, whereas m-PEG–TFSI^–^ is only 0.23 eV stronger than the m-PFPE–TFSI^–^ relaxed coordination state. The higher stability of both Li^+^ and TFSI^–^ in the PEG phase, as compared to the PFPE phase, drives the creation of an ion-rich transport domain for Li^+^ conduction via phase separation of the salt into PEG, as supported by the spectroscopic behavior of TFSI^–^ and Li^+^ in Figure 3c,d.

### Ion Mobility and Transport Behavior

As observed in typical polymer-in-ceramic, ceramic-in-polymer, and multiphase polymer systems, ion transport typically takes place in the phase with the lowest resistance to charge transfer.^33,42,43^ It is clear that the salt resides primarily in the PEG phase, which is the expected ion transport domain through the nonconductive PFPE matrix. This is apparent both on the nanoscale, using spin–lattice relaxation time (*T*_1_) measurements in MAS NMR, and on the microscale, through the fitting of the conductivity as a function of temperature using the Vogel–Tammann–Fulcher (VTF) equation to obtain the activation energy (*E*_a_) to ionic conduction shown in Figure 4a.

Spin–lattice relaxation time (*T*_1_) is a measure of how quickly energy is transferred from the excited Li nuclei to its surroundings. This parameter captures the local mobility of Li^+^ within the polymer matrix, as vibrational interactions causing movement will lead to energy transfer from Li^+^ to the surrounding lattice.^22,44−46^*T*_1_ quantifies the characteristic timescale of nanoscale lithium-ion motion as it jumps between coordination states and transfers its energy to the polymer matrix after the initial radio wave excitation. This experiment was conducted on each polymer sample including the PFPE and PEG controls and all five PEG/PFPE ratios with a salt content of *r* = 0.05 LiTFSI. Experimental details are outlined in the Supporting Information, with the pulse sequence shown in Figure S13. Figure 4a shows that *T*_1_ for the pure PFPE sample is about 3 s, while *T*_1_ for all of the polymer ratios and for pure PEG is around 0.5 s. This provides clear evidence that the mechanism of ion transport in the polymer composite samples is the same as the mechanism in pure PEG and confirms that ion transport is confined to the PEG phase. The large difference in *T*_1_ times between Li^+^ in PFPE and Li^+^ in the composite and PEG samples highlights the frequency with which Li^+^ interacts with the polymer environment in each case. For Li^+^ in PFPE, the *T*_1_ relaxation time is 6 times higher than *T*_1_ in the other samples.

Despite the similarity of the ion transport mechanism between the composite polymer samples, there remains a large difference in conductivity stemming from the varying phase morphology as the PEG/PFPE ratio changes, as outlined in Figure 1b. This can be quantified by analyzing the conductivity as a function of temperature with a VTF fit. Figure 4a shows both the VTF activation energy and the PEG phase *T*_g_ measured with DSC, which was utilized in the VTF fitting for each polymer. Fitting the conductivity curves with the *T*_g_ of the PEG phase in each composite sample was found to produce the best fitting results, as compared to fittings using the PFPE phase *T*_g_ in Figure S14. For this reason, the *T*_g_ of interest for ion conduction is the PEG phase *T*_g_, and Figure 4a presents this value for each of the composite polymers. The *T*_g_ value at EO/(EO + CF_2_) = 0 represents the pure PFPE *T*_g_, explaining the large difference in magnitude.

The activation energy (*E*_a_) correlates with the *T*_g_ data, where the higher *T*_g_ in the 1P3F and 1P1F polymers (lowest EO mole ratios) correlates with higher *E*_a_. The increase in *E*_a_ from the higher PEG content polymers to the 1P3F and 1P1F polymers is much larger than the increase in *T*_g_, but it is reflective of the PEG phase constriction, as the PFPE phases become larger and denser within the PEG matrix. When the PEG channels are more constricted, edge effects likely become a large aspect of ion transport inhibition, where ions will prefer to transfer in the PEG bulk domain toward the center of the channels.^24,33,47^

Combined *T*_1_ and *E*_a_ analysis gives insight into the importance of both nano- and microscale processes, as the segmental motion mechanism can be interrupted by microscale factors such as phase interfaces and end-to-end (electrode-to-electrode) path convolution. Together, we can see that NMR shows the average random fluctuations and movement of Li^+^ through the ideal PEG solvation environment, but when electrochemical polarization is applied across the polymer film in a stainless steel|stainless steel (SS|SS) cell, the directed movement of Li^+^ through the film becomes disrupted by the phase boundaries and PEG phase convolution. Thus, the ion mobility becomes inhibited on the microscale and trends directly with the amount of PEG present.

### Effect of Polymer Mobility on Ion Mobility and Application in Battery Systems

Interestingly, the DSC analysis in Figure 4a shows that the PFPE sample *T*_g_ is −108 °C, an order of magnitude lower than the *T*_g_ of the pure PEG and of the PEG phase in each composite sample, which is approximately −45 °C on average. Typical polymer electrolyte studies show that lower *T*_g_ correlates directly with higher conductivity, but here, we observe a counter example. Notably, PFPE is a very tough film once cross-linked. Due to the symmetrical methacrylate end groups on the PFPE chains, the cross-link density within the pure PFPE solid polymer is high with 1500 g/mol between each cross-link. Therefore, the low *T*_g_ promotes polymer mobility at low temperatures, but the short distance between cross-linking points restricts the segmental motion of each section of the polymer chain to the region between its cross-linking points. In contrast, the pure PEG sample has no cross-linking points, so the bottlebrush chains exhibit unrestricted segmental motion, promoting fast transport of the solvated Li ions but diminishing the material strength. Dynamic mechanical analysis (DMA) was used to observe the differences in polymer viscoelasticity among the PFPE, PEG, and PEG/PFPE (3:1) (3P1F) samples. Frequency sweep experiments were performed at −50 °C, which is near the PEG *T*_g_, and at 25 °C.

At −50 °C, in Figure 4b, the 3P1F and PEG samples exhibit the transition from amorphous to crystalline states, evidenced by the slope in the storage and loss moduli, where the amorphous state has lower moduli and the crystalline state has higher moduli. The slope of the 3P1F sample is less steep, showing the strengthening effects of PFPE inclusion within the PEG matrix in the polymer composite samples.^48^ At −50 °C, crosslinked PFPE is in its amorphous state; yet, it still exhibits a storage modulus higher than that of PEG for all frequencies and higher than 3P1F until the frequency exceeds 10 rad/s. At room temperature, in Figure 4c, all of the polymers are in their amorphous states. Crosslinked PFPE shows the highest storage and loss moduli with 3P1F and PEG following, in decreasing order. The separation between the storage and loss moduli in PFPE and 3P1F shows the prominence of the solid-like behavior in these polymers. The PEG sample shows a highly viscoelastic behavior at room temperature with nearly equivalent storage and loss modulus values.

These results show that PEG has high mobility and will flow to form good interfaces with contacting surfaces. The high modulus of PFPE restricts it from flowing, leading to low ability to adhere to surfaces despite its low *T*_g_ and nanoscale flexibility between cross-links. The composite 3P1F sample balances the mechanical behavior of the two pure polymers and exhibits moderate storage and loss moduli. This grants the film both high strength for ease of processability and cell fabrication as well as good viscoelasticity to promote contact at electrolyte|electrode interfaces. Additionally, the enhanced polymer mobility supports long-distance ion transport, as polymer segmental mobility is not constrained between cross-linking points in the PEG phase.

### Electrochemical Stability at Lithium Metal and Cathode Interfaces

**Figure 5:**
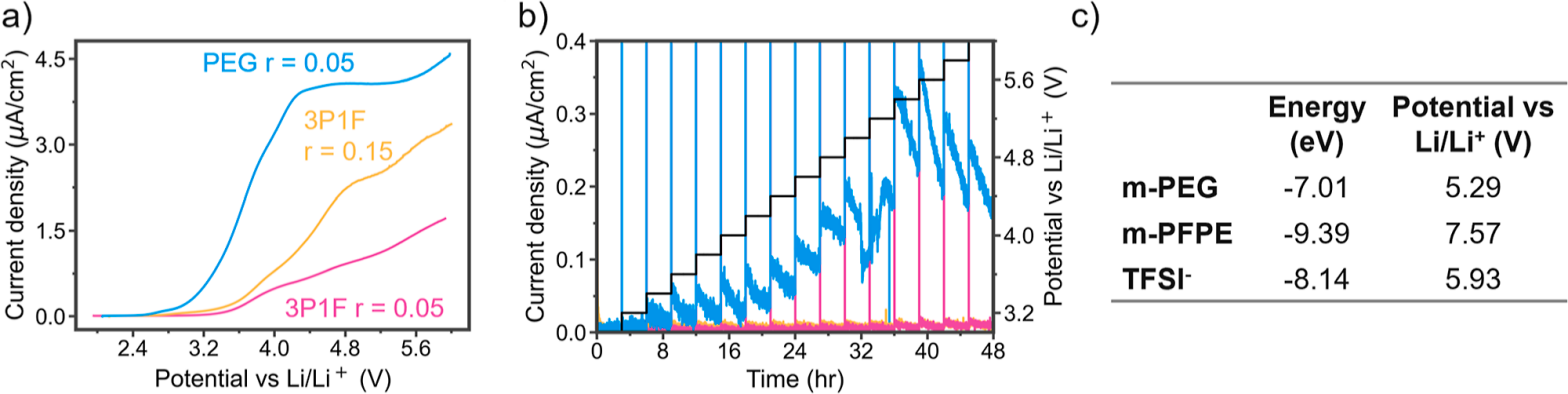
High-voltage stability. (a) LSV with 1 mV/s scanning rate for Li|Al cells and (b) potentiostatic hold experiments for Li|Al cells with the 3P1F *r* = 0.05, 3P1F *r* = 0.15, and PEG *r* = 0.05 polymer electrolytes. (c) Highest occupied molecular orbital (HOMO) energy levels and corresponding oxidation potentials vs Li/Li^+^ for the m-PEG chain, m-PFPE chain, and TFSI^–^ molecule derived from DFT energy calculations for the relaxed structures in their initial and oxidized states.

The composite *x*P*y*F system succeeds at (1) introducing ionic conduction to the fluorinated ether system with conductivity through the PEG ion channels on the order of that in pure PEG (Figure S2) and (2) modulating the polymer mobility to support ion transport and ideal viscoelasticity for interface formation at the electrode surfaces. Now, it is essential to prove that the system retains the heightened stability of the fluorinated entities and supports reversible Li metal plating and stripping at the lithium metal anode. The stability of the polymer samples was investigated via linear sweep voltammetry (LSV) and potentiostatic hold experiments using Li|Al (Figure 5) and Li|SS cells (Figure S15). The reversibility of Li metal plating and stripping was tested in Li–Li symmetric cells.

Oxidative stability testing was performed on the PEG *r* = 0.05, 3P1F *r* = 0.05, and 3P1F *r* = 0.15 electrolytes to compare the effects of fluorination from both the PFPE phases and the TFSI anion on the electrochemical stability. Experiments were conducted by using both aluminum and stainless steel working electrodes to investigate the electrochemical stability of the electrolytes against various interfaces. Electrochemical stability in Li|Al and Li|SS cells is shown in Figures 5 and S15, respectively. The LSV experiments displayed in Figure 5a show the stability of the electrolyte against an Al working electrode as the potential is increased to 6 V. These data are highly reproducible, as shown in Figure S16. The voltammograms do not show appreciable oxidation currents for the three polymer samples, likely due to mass transfer limitations and slow kinetics at the solid–solid interface in the tough polymer electrolytes.^49,50^ This makes it difficult to probe exact oxidation potentials from the LSV curves, as oxidative currents around 10–50 μA/cm^2^ are typically used as cutoff values and the high diffusion limitation increases the error on observable features.^36,50−52^ Despite the implications of mass transport limitations, consistent differences in the magnitude of the Faradaic currents between the three samples give valuable insights into the effects of the inclusion of PFPE and LiTFSI. The PEG control demonstrates the highest Faradaic current, followed by the 3P1F *r* = 0.15 LiTFSI and, finally, the 3P1F *r* = 0.05 LiTFSI samples. This trend reveals that the addition of PFPE enhances the stability of the polymer against oxidation. LiTFSI is known to degrade at electrode interfaces to form passivating degradation layers, and the LSV shows this degradation with an enhanced Faradaic current in the 3P1F sample with a higher salt content. This shows the importance of both the salt and the fluorinated backbone in stabilizing the electrolyte for the phase-separated morphology.

The potentiostatic hold experiments in Figure 5b give a more rigorous analysis of the oxidation potential by exposing the samples to high potentials at 3 h increments. The slow stepwise process yields better approximations of the oxidation potential, with the current threshold set to 0.1 μA/cm^2^. The PEG control shows appreciable oxidation at 4.6 V, with the Faradaic current lying fully above the current threshold at 4.8 V, which aligns well with the recent reports of PEO samples.^53^ The addition of PFPE stabilizes the composite 3P1F polymer with *r* = 0.05 and *r* = 0.15 LiTFSI beyond 6 V, enhancing the oxidative stability significantly when compared to the pure PEG samples.

Measurements of oxidative stability against Al and SS working electrodes likely do not capture the full extent of reactions that the electrolyte undergoes in a full battery cell. Lithium iron phosphate (LiFePO_4_, LFP) cathodes were used as working electrodes to investigate the electrolyte’s stability in the presence of chemical interactions at the cathode|electrolyte interface as the potential was increased, as shown in Figure S17. In potentiostatic hold testing, Figure S17a, all the samples exhibit peaking features, starting at 3.8 V for PEG and around 4.2 V for the 3P1F electrolytes. This peak can be correlated with lithium deintercalation. The deintercalation of Li from LFP occurs between 3.5 and 3.6 V;^54−56^ however, here, we see delayed deintercalation due to the high overpotential to interfacial electrochemical reactions in the polymer electrolytes. After the deintercalation reactions complete, the Faradaic current decreases to a baseline value, which can be attributed to continuous degradation. The PEG *r* = 0.05 sample shows the highest degradation beyond 5.6 V, followed by 3P1F *r* = 0.15 and 3P1F *r* = 0.05. LiTFSI is known to continuously oxidize at LFP leading to cell failure, and this added chemical aggravation at the non-blocking LFP electrode is likely the main cause for the difference in oxidative stability between the high and low salt loading 3P1F samples.^57,58^

Galvanostatic charging of the 3P1F *r* = 0.05 LiTFSI sample with a 6 V cutoff voltage was also conducted, as shown in Figure S17b, to observe the impact of continued oxidizing current after full charging of the cell. The three replicates show that the delithiation reaction occurs between 3.6 and 4.4 V, after which the resistance to further delithiation is too high, leading to continued voltage increase and subsequent electrolyte degradation above 4.7 V. Due to the high overpotential to cycling, the high cutoff voltage allows for an order of magnitude increase in accessible capacity as compared to cycling attempts seen in Figure S20, which have a cutoff voltage of 3.8 V.

The HOMO (highest occupied molecular orbital) energy levels and oxidation potentials obtained using DFT in Figure 5c dictate the theoretical stability ranking of the polymers and the anion. These calculations are in good agreement with other reports, as shown in Table S1. The oxidation potentials rank with m-PFPE highest at 7.57 V, the dissociated TFSI^–^ second highest at 5.93 V, and m-PEG weakest at 5.29 V. The lower oxidative stability of TFSI^–^ as compared to m-PFPE highlights the prevalence of TFSI^–^ contribution to the cathode electrolyte interface and explains the early rise in oxidative current observed in the LSV experiments in Figure 5a. The heavy fluorination of PFPE was desired to heighten the oxidative stability of the polymer system, but due to the phase separation, TFSI^–^ is an important sacrificial component of the electrolyte.

**Figure 6:**
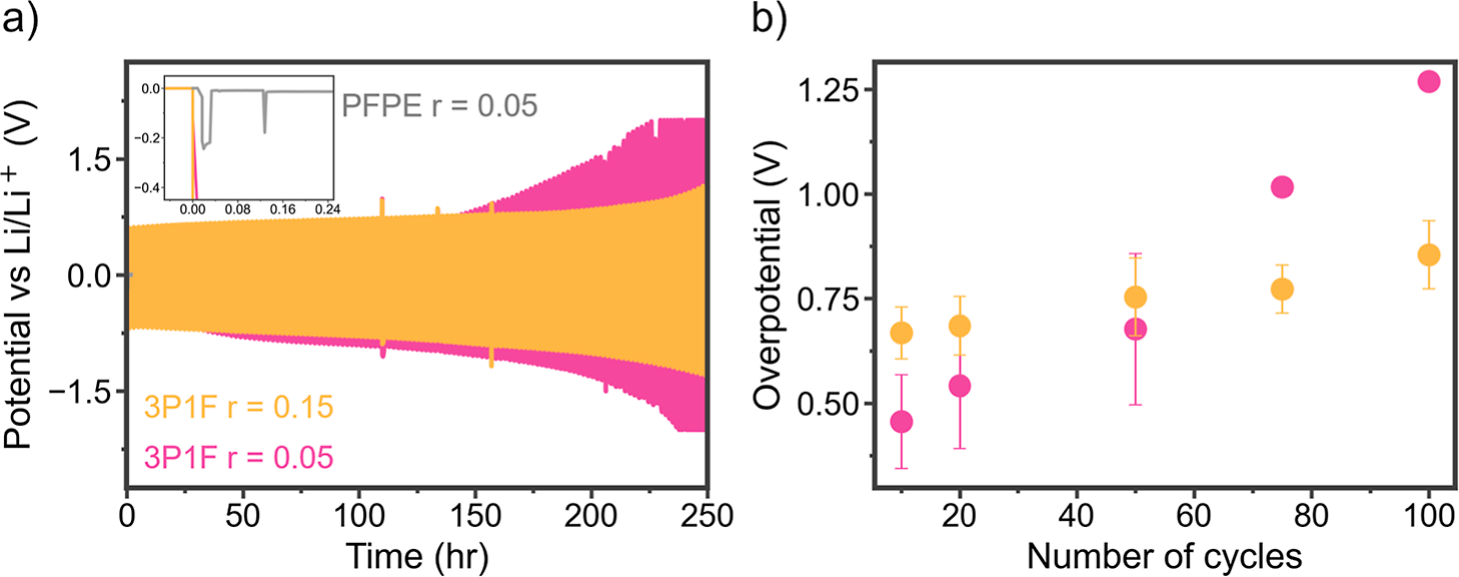
Electrochemical performance. (a) Li|Li cycling at 0.1 mA/cm^2^ for 3P1F *r* = 0.05, 3P1F *r* = 0.15, and PFPE *r* = 0.05, with inset showing the immediate shorting of the PFPE sample. (b) Analysis of the overpotential of Li stripping for 3P1F with *r* = 0.05 and *r* = 0.15 throughout the first 200 h of cycling. All points have error bars representing three samples except for the points at 75 and 100 cycles for the 3P1F *r* = 0.05 cell, as one of the three cells died after 70 cycles. These two points represent an average of two coin cells.

Finally, Li|Li cycling was performed at 0.1 mA/cm^2^ to demonstrate the reversibility of Li plating and stripping in the 3P1F samples. Previous studies of PFPE solid polymer systems have not shown successful full cell or symmetric cell cycling.^21,22^ As shown in Figure 6a, the addition of PEG side chains into PFPE allows for the reversible plating and stripping of lithium in Li|Li cells for over 200 h. The potential profiles in Figure S18 show flat plateaus for the 3P1F *r* = 0.15 sample, while the 3P1F *r* = 0.05 sample shows an arched profile. This arched profile has been shown to correlate with diffusion-limited processes at the lithium metal interface stemming from tortuous deposits of dead lithium and patchy, uneven SEI formation.^59^ The *r* = 0.05 polymer likely has higher organic content in its degradation layers leading to less uniform, continuously growing interfacial layers, causing the arching voltage profile.^59−61^ The PFPE *r* = 0.05 electrolyte did not support Li|Li cycling as the rigid polymer could not form a good interface with lithium metal to support electrochemical reactions at the solid–solid interface, and the cell quickly shorted after the onset of the experiment. Four attempts to cycle the Li|Li cell with PFPE electrolyte are shown in Figure S19, all of which could not start due to poor interfaces despite long-term heating at high temperatures.

The overpotentials of Li stripping in the 3P1F samples over the course of the 250 h of cycling are shown in Figure 6b. The overpotential to lithium plating and stripping is high at 500 mV as compared to overpotentials at or below 100 mV in polymer systems operated in the melt or with low degree of polymerization plasticizers.^6,29,62^ The pure PEG *r* = 0.05 LiTFSI sample reflects this literature with an overpotential well below 100 mV in Figure S18. Introduction of cross-linking and other techniques to increase mechanical integrity without the addition of plasticizers tends to report Li|Li cycling with a high overpotential on the order of several hundred mV as demonstrated in our cross-linked polymer blend in Figure 6b.^63,64^ The ability to enable electrochemical reactions at the solid–solid interface with a high storage modulus material is notable; however, the rigidity of the PFPE phases restricts improved interfacial dynamics, and polymer mobility is an important design factor for future electrolytes.

The 3P1F *r* = 0.05 sample exhibits a linear increase in overpotential, while the 3P1F *r* = 0.15 sample exhibits a relatively constant overpotential. The high salt content polymer initially has a higher overpotential due to the lower conductivity in this system (Figure S2). However, after initial SEI-forming reactions consume LiTFSI in each system, it is possible that a higher concentration overpotential exists in the *r* = 0.05 sample as compared to the *r* = 0.15 sample, as the lower concentration of salt leads to enhanced mass transport limitations, while the salt diffuses to the ion-depleted interface.^59,65^ Overpotential likely also increases more rapidly in the *r* = 0.05 sample due to hindered diffusion through the organic heavy degradation layers, as discussed previously based on the arched potential profile (Figure S18).

The high overpotential for Li|Li cycling was found to heavily impact cycling in Li|LFP cells, as discussed in the Supporting Information with Figure S20. The good mobility of the PEG phase formed direct contact between the LFP particles and the polymer to initially promote electrochemical reactions at the interface; however, the high overpotential due to the PFPE phase area causes the cell to quickly meet its cutoff voltage and exhibit impractical discharge capacities. Future polymer electrolyte designs should focus on increasing the backbone mobility and miscibility of fluorinated and ether components to promote conductivity through the bulk, oxidative stability across the full polymer surface area, and ultimately enable better cycling capacities.

The LSV, potentiostatic hold experiments, DFT, and Li|Li cycling show that both PFPE and LiTFSI contents work together to increase the oxidative stability of the composite polymer. The cross-linked polymer blend successfully combines the ion transport properties of PEG with the mechanical integrity and high oxidative stability of the PFPE component. No PFPE-based electrolyte in the literature has shown the ability to cycle. Here, we see that the engineering of PEG microchannels within the PFPE bulk provides pathways for electrochemical reactions at solid|solid interfaces between soft PEG and the electrodes while maintaining the beneficial aspects of pure PFPE. This polymer blend still shows a high overpotential to cycling due to the restriction of mobility at cross-linking points; however, the ability to plate and strip lithium metal and resist appreciable oxidation above 6 V highlights that this electrolyte design approach is promising for implementation in high energy density battery applications.

## Conclusions

The blending of fluorinated and ether macromolecules to form a cross-linked copolymer network yielded the successful enhancement of both ionic conductivity and oxidative stability in the solid-state polymer electrolyte. The fluorinated ether polymer blend electrolyte has 6 orders of magnitude difference in conductivity as compared to pure PFPE from 1.55 × 10^–11^ to 2.26 × 10^–5^ S/cm at 60 °C and exhibits >6 V oxidative stability as compared to 4.6 V oxidative stability limit in pure cross-linked PEG electrolyte against aluminum. This system can reversibly plate and strip lithium, showing good contact at the lithium interface.

The study of ion solvation and mobility in the ternary PEG–PFPE–LiTFSI system revealed the strong affinity of the hydrophilic salt for the PEG phase. The strong electron-withdrawing effect of CF_2_ groups in PFPE led to poor electron density across the backbone, making binding of Li^+^ and TFSI^–^ to the PFPE backbone weak and unfavorable. Via transport analysis, it is apparent that the Li ions exhibit single-phase transport through the PEG domain. At the nanoscale, the varying PEG/PFPE ratio films show no difference in ion hopping dynamics via MAS NMR analysis; however, on the microscale, the presence of phase boundaries inhibits ion motion due to more tortuous PEG pathways in low PEG content films, causing higher activation energies and lower conductivities. The ion transport is inherently tied to the phase morphology of the polymers, and optimization of the phase separation in the 3P1F film showed conductivities comparable to pure PEG. Ideally, a lower fluorinated density in the polymer network could enable enhanced blending of ether and fluorinated components to promote the inclusion of high-stability moieties within the dominant ion transport phase. This system showcases the exceptional potential of solid-state fluorinated ether polymer electrolytes and highlights the importance of ion–polymer interactions in the design of new polymer systems.

## Methods

### *x*P*y*F (PEG/PFPE (*x*/*y*)) Polymer Fabrication

The cross-linked polymers were fabricated from methacrylate-functionalized PEG and PFPE macromonomers in an argon-filled glovebox (Vigor, O_2_ and H_2_O < 1 ppm). Methyl methacrylate PEG (M_n_ = 500, Sigma-Aldrich) and PFPE dimethacrylate (Fluorolink MD700, M_w_ = 1500 g/mol, Solvay) were added to a dry vial containing *r* = 0.05 [Li^+^/EO ratio] LiTFSI (99.95% trace metals basis, Sigma-Aldrich). The *x*P*y*F class of polymers has an *x*/*y* molar ratio of PEG chains to PFPE chains. For example, the 3P1F sample has three PEG chains for every 1 PFPE chain. The salt ratio is only with respect to the EO units in the PEG chains for polymer composite samples. Example calculations for PEG/PFPE and salt contents in the 3P1F film are discussed in the Supporting Information. Tetrahydrofuran (THF, anhydrous, >99.9%, inhibitor-free, Sigma) was dried on molecular sieves inside a glovebox and was added to this mixture in a ratio of 1.2 μL THF/mg LiTFSI. After mixing for 15 min, 5 wt % azobis(isobutyronitrile) (AIBN, 98%, recrystallized in methanol, Sigma-Aldrich) with respect to the total polymer mass was added, and the mixture was left to mix for 5 more minutes. This thin, cloudy mixture was cast between two glass plates (4 × 4 × 1/8 in., Fisher) with 100 μm microscope slide spacers to control the polymer thickness. The film was left on a hot plate in the glovebox at 70 °C to cure for 1 h. Subsequently, the polymer films were dried overnight at 70 °C under vacuum to remove trace amounts of THF. After drying, NMR was used to confirm the absence of THF in the films. No residual THF was observed.

The PEG and PFPE control samples were fabricated by the same method. The PEG control sample was cast on fluorinated ethylene propylene sheets (FEP, 0.1 mm thick, Outus) between the glass plates. The FEP sheets easily transferred the low mechanical strength PEG sample onto other surfaces for characterization and testing.

### SEM Analysis

The phase separation and polymer morphology were investigated by using a field emission scanning electron microscope (Carl Zeiss Merlin). Prior to testing, the samples were sputter-coated with a 5 nm platinum/palladium coating. Sulfur distribution was mapped with an Oxford UltimMax100 EDX spectroscopy sensor. The applied voltage was 10 kV with a working distance of 8.5 mm.

### DSC Analysis

DSC measurements of the PFPE and PEG phase glass-transition temperatures were taken on a Mettler-Toledo DSC823e at the Argonne National Laboratory Center for Nanoscale Materials. Each polymer sample was loaded into a Tzero pan with a hermetic lid and crimped inside of an argon-filled glovebox. To capture both glass-transition temperatures, the thermal program ran from −150 to 0 °C for three cycles at a ramp rate of 10 °C/min. The instrument was equipped with a liquid nitrogen cooling line to cool the system to −150 °C.

### Raman Spectroscopy

Salt dissociation was probed with a HORIBA LabRAM HR evolution confocal Raman microscope. The spectra were centered on 930 cm^–1^ with a 600 gr/mm grating and were collected in 32 accumulations of 5 s with a 532 nm ultralower frequency laser. The samples were sealed onto a microscope slip inside an argon-filled glovebox using microscope glass coverslips and silicone isolators from Grace Bio-Laboratories.

### Nuclear Magnetic Resonance Spectroscopy

^7^Li and ^19^F MAS NMR spectra were obtained on a Bruker Avance III wide-bore 400 MHz solid-state NMR spectrometer with a 9.5 T field. The samples were packed into 1.9 mm zirconia rotors (Bruker) in an argon-filled glovebox and spun at 20 kHz. The ^7^Li spectra were referenced to solid LiF at −1.0 ppm with a reference frequency of −378.24 Hz, and the signal was tuned to a Larmor frequency of 155.5 MHz. The spectra were collected after a 5 s relaxation delay following a 90 W, 0.9 μs 90-degree pulse. Each spectrum is an average of eight scans. Li^+^ T_1_ was calculated from a set of 12 collections with varying delay times. The target peak intensity over the 12 runs was fit to an exponential function to find the *T*_1_ value; more details can be found in the Supporting Information with Figure S13. The ^19^F spectra were referenced to LiF at −203 ppm with a reference frequency of −1742.54 Hz, and the signal was tuned to a Larmor frequency of 376.6 MHz. The spectra were collected after a 5 s relaxation delay following a 4.2 W, 4 μs 90-degree pulse. Each spectrum is an average of 32 scans.

### DFT Calculations

Binding energy, ionization energy, and HOMO–LUMO energy level calculations were conducted with Gaussian (Version 16 Revision A.03) using the B3LYP functional and 6-31G**(d,p) basis set.^39^ Representative PEG and PFPE macromonomers (m-PEG and m-PFPE) were constructed with nine and five EO units, respectively. m-PEG is of the same length as the PEG methacrylate macromonomer used to synthesize the *x*P*y*F polymers; however, m-PEG does not have a methacrylate end group. m-PFPE contains 5 EO groups compared to 10 EO groups in the PFPE macromonomer used in the *x*P*y*F polymers. The EO groups are a random sequence of (CF_2_O) and (CF_2_CF_2_O) units as in PFPE, where m-PFPE contains a 2:3 ratio of (CF_2_O)/(CF_2_CF_2_O) to mimic the 3:7 ratio in PFPE. m-PFPE does not include the methacrylate and amine components of the end groups in PFPE. These simplifications were made to minimize the computational time for the calculations. The molecular visualizations were generated using visual molecular dynamics software (version 1.9.4).^66^

The single segment m-PFPE and m-PEG were first relaxed in a vacuum with and without the Li^+^ and TFSI^–^ ions. The same relaxation calculations were then performed on the systems with implicit solvation using the polarizable continuum model,^67^ where ε_PEG_ = 7.5 and ε_PFPE_ = 2.1, to calculate the final binding and system energies.^68^ A radius of 3.25 Å was employed to identify coordinating atoms between the ion and polymer segments, where the strongest interactions occurred <3 Å. System energies of neutral (negative for TFSI^–^) and positively charged structures (neutral for TFSI^0^) were used to calculate the ionization energy upon oxidation, which was converted to the Li/Li^+^ scale by subtracting 1.4 to obtain the oxidation potential.^69,70^

### Dynamic Mechanical Analysis

DMA was performed on an RSA-G2 dynamic mechanical analyzer (TA Instruments). Amplitude sweep experiments were performed at 6.28 rad/s (1 Hz) to identify the maximum strain value within the elastic regime of each sample. This strain value was then used in frequency sweep experiments from 1 to 100 rad/s. An air chiller system was used to analyze samples below room temperature.

### Electrochemical Impedance Spectroscopy

EIS was carried out on a Biologic VSP-300 potentiostat with a frequency range from 7 MHz to 1 Hz and a voltage amplitude of 10 mV. EIS was conducted at 11 temperatures from 120 to 20 °C, with the temperature controlled by an ESPEC environmental chamber (BTZ-133). The coin cell samples were prepared inside an argon-filled glovebox with stainless steel, ion-blocking electrodes. To prepare a coin cell, the polymer sample was punched into an 11 mm disk and sandwiched between two stainless steel disks. Celgard rings with a 12 mm inner diameter were used to inhibit contact between the edges of the stainless steel electrodes. The AC conductivity was calculated from the average of the plateau in the real conductivity Bode plot. This value is consistent with the conductivity approximated by fitting a Randles circuit to the Nyquist impedance plots. The Bode plot analysis yielded a lower error in the conductivity calculation. The comparison of the Bode plot and Nyquist plot analysis methods is outlined in Figure S21.

### LSV/Potentiostatic Hold Experiments

The oxidative stability of the polymer samples was tested with LSV and potentiostatic hold experiments on a Biologic MPG-2 potentiostat. Li|Al and Li|SS coin cells were prepared in a glovebox for LSV experiments. Prior to testing, the coin cells were rested for 12 h at 80 °C in a Memmert IN 110 oven to ensure a good interface was formed at the polymer|electrode interface. An annealing test was performed to confirm that the polymer phase morphology does not change during this pretest annealing step and is shown in Figure S22. Starting at an open-circuit voltage (OCV), the potential was scanned to 6 V at a scan rate of 1 mV/s. Li|Al, Li|SS, and Li|LFP coin cells were prepared in a glovebox for potentiostatic hold experiments. Again, the cells were held at the OCV in an 80 °C oven for 12 h before testing. The procedure held the sample at potentials from 3 to 6 V versus Li/Li^+^ for 3 h increments. Each step increased the potential by 0.2 V for a total of 15, 3 h potential steps. LFP electrodes were obtained from the Cell Analysis, Modeling, and Prototyping (CAMP) facility at the Argonne National Laboratory. The cathode slurry contained 92 wt % active material and was coated onto an aluminum foil with a thickness of 66 μm. The areal capacity is 1.88 mAh/cm^2^.

### Li|Li Cycling

Li|Li symmetric coin cells were prepared to test the reversibility of Li plating and stripping at the polymer|Li interface. The Li|Li cycling was performed using a Neware BTS4000 battery tester at 80 °C in a Memmert IN 110 oven. The cells were rested at OCV for 12 h in the oven before cycling. After the rest step, the cells were cycled at 0.1 mA/cm^2^ with 2 h charge and discharge steps. The cut off voltages were set to −2 and 2 V versus Li/Li^+^.

## Supporting Information Available

Detailed polymer fabrication calculations, SEM morphology studies with salt loading, conductivity analysis with varying electrolyte formulations, EDX spectra and mapping of 3P1F electrolyte, phase morphology–conductivity correlation analysis, detailed DSC thermogram and analysis for each polymer blend and polymer control, raw and processed Raman spectroscopy, raw MAS NMR spectra for all polymer blends and control samples, *T*_1_ experiment pulse program and method, conductivity fitting technique, LSV, potentiostatic hold, and galvanostatic charge experiment details for Li|Al, Li|SS, and Li|LFP cells, Li|Li cycling for all PEG, 3P1F and PFPE electrolytes, Li|LFP cycling for 3P1F *r* = 0.05 electrolyte, EIS data analysis technique, analysis of effect of high-temperature annealing on phase morphology, and cross-validation of oxidative stability calculations with literature results (PDF)
