# Supplementary material for: Phase Morphology Dependence of Ionic Conductivity and Oxidative Stability in Fluorinated Ether Solid-State Electrolytes
Source: Chem Mater. 2024 May 9;36(10):5063–76. doi: 10.1021/acs.chemmater.4c00199 (PMC11137829; doi:10.1021/acs.chemmater.4c00199)
Supplement: Supplementary file 1 — cm4c00199_si_001.pdf [file cm4c00199_si_001.pdf]

Supporting Information

Phase Morphology Dependence of Ionic Conductivity and Oxidative Stability in Fluorinated Ether Solid-State Electrolytes

Emily S. Doyle,[1] Priyadarshini Mirmira,[1] Peiyuan Ma,[1] Minh Canh Vu,[1] Trinity Hixson-Wells,[1] Ritesh Kumar,[1] and Chibueze V. Amanchukwu[1*]

[1]Pritzker School of Molecular Engineering, University of Chicago, Chicago IL 60637, United States

*Corresponding author (chibueze@uchicago.edu)

Methods

Phase composition was determined by the following calculations done for the 3P1F blend:

1. **PEG:PFPE ratio**
   3P1F → 3 PEG chains : 1 PFPE chain
   MW_{PEG} = 500 g/mol ; MW_{PFPE} = 1500 g/mol
   500 g/mol x 3 mol = 1500 g PEG
   1500 g/mol x 1 mol = 1500 g PFPE

   For a batch using 0.2 g PEG:
   $$0.2\ g\ PEG \cdot \frac{1500\ g\ PFPE}{1500\ g\ PEG} = 0.2\ g\ PFPE$$

2. **Absolute salt content (r=0.05)**
   r = Li^{+} / Ether oxygen unit (EO) = 0.05

   For the polymer blend samples, only the PEG EO groups were counted in the calculation, since EO groups in PFPE are guarded by CF_2 groups that weaken the EO groups' abilities to coordinate Li^{+}.
   1 PEG chain has 9 EO groups

$$0.2\ g\ PEG \cdot \frac{mol\ PEG}{500\ g\ PEG} \cdot \frac{9\ EO}{PEG\ chain} \cdot \frac{N_{AV}\ PEG\ chains}{mol\ PEG} \cdot 0.05\ \frac{Li^{+}}{EO} \cdot \frac{mol\ Li^{+}}{N_{AV}\ Li^{+}} \cdot \frac{287.09\ g\ LiTFSI}{mol\ Li^{+}}$$

   = 0.052 g LiTFSI for 3P1F r = 0.05 sample with 0.2 g PEG and 0.2 g PFPE

   For pure PFPE, the # EO units in the PFPE chain are used despite their low ability to coordinate Li^{+}.

   1 PFPE chain has 10 'EO' groups

$$0.2\ g\ PFPE \cdot \frac{mol\ PFPE}{1500\ g\ PFPE} \cdot \frac{10\ EO}{PFPE\ chain} \cdot \frac{N_{AV}\ PFPE\ chains}{mol\ PFPE} \cdot 0.05\ \frac{Li^{+}}{EO} \cdot \frac{mol\ Li^{+}}{N_{AV}\ Li^{+}} \cdot \frac{287.09\ g\ LiTFSI}{mol\ Li^{+}}$$

   = 0.019 g LiTFSI for PFPE r = 0.05 sample with 0.2 g PFPE

3. **AIBN and THF calculations**
   For the 3P1F r = 0.05 LiTFSI film:

   $$\frac{1.2\ \mu L\ THF}{mg\ LiTFSI} \cdot 52\ mg\ LiTFSI = 62.4\ \mu\text{L THF}$$

   5 wt% AIBN with respect to the total polymer mass was added to the PEG, PFPE, LiTFSI, THF solution:
   0.2 g PEG + 0.2 g PFPE = 0.4 g polymer *0.05 = 0.02 g AIBN

Supplementary Figures

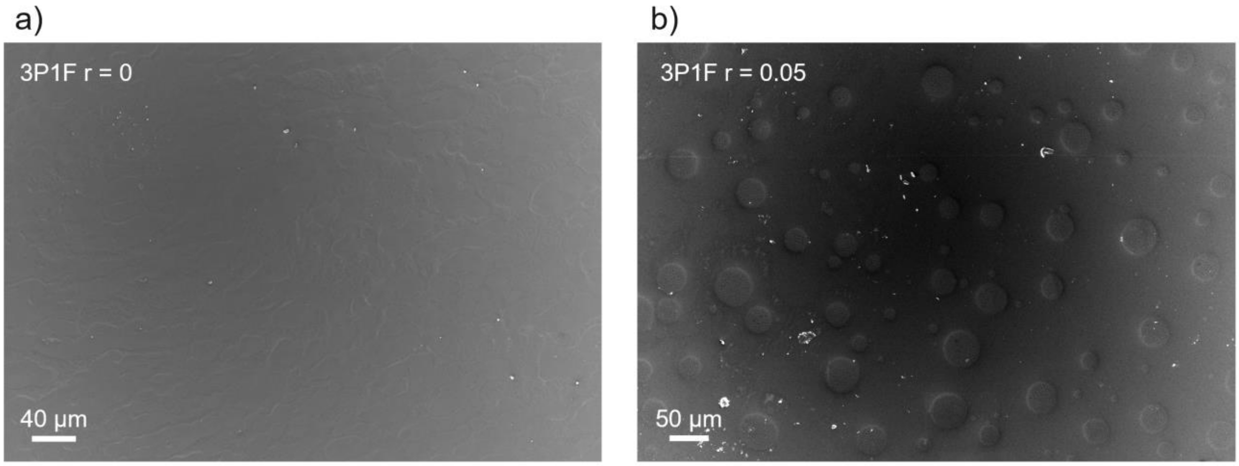

Figure S1. SEM images showing variation in surface phase morphology in 3P1F r = 0 and r = 0.05 films.

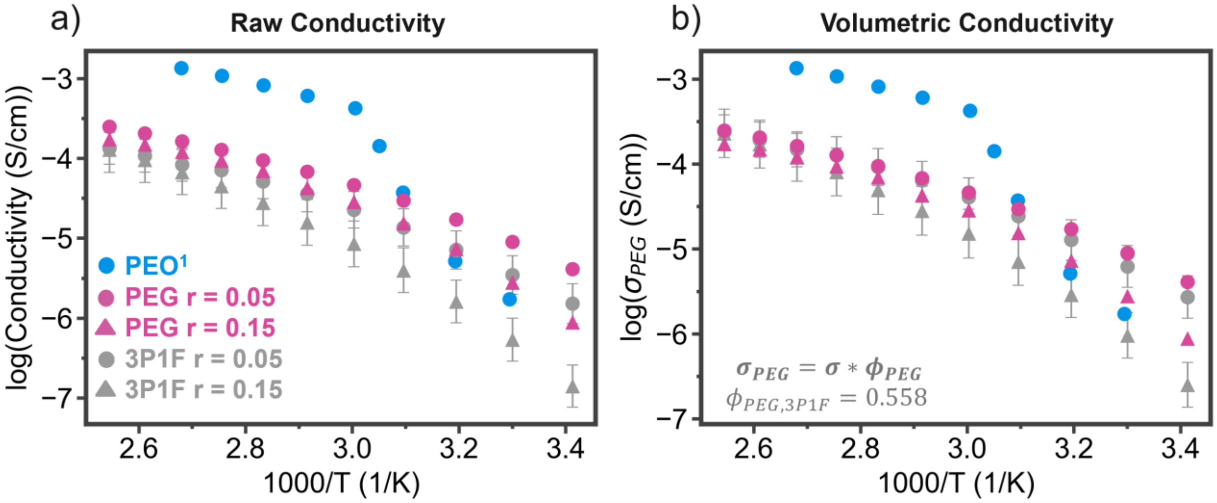

Figure S2. a) Raw conductivity data for PEO[1], PEG and 3P1F samples as compared to **b)** volumetric conductivity, where the 3P1F sample conductivity is normalized by the PEG volume fraction. This normalization shows equivalent conductivity of the PEG electrolyte and the PEG phase in the 3P1F electrolyte. The PEO data was extracted from work by Devaux et al.[1] using WebPlotDigitizer version 4.7.[2]

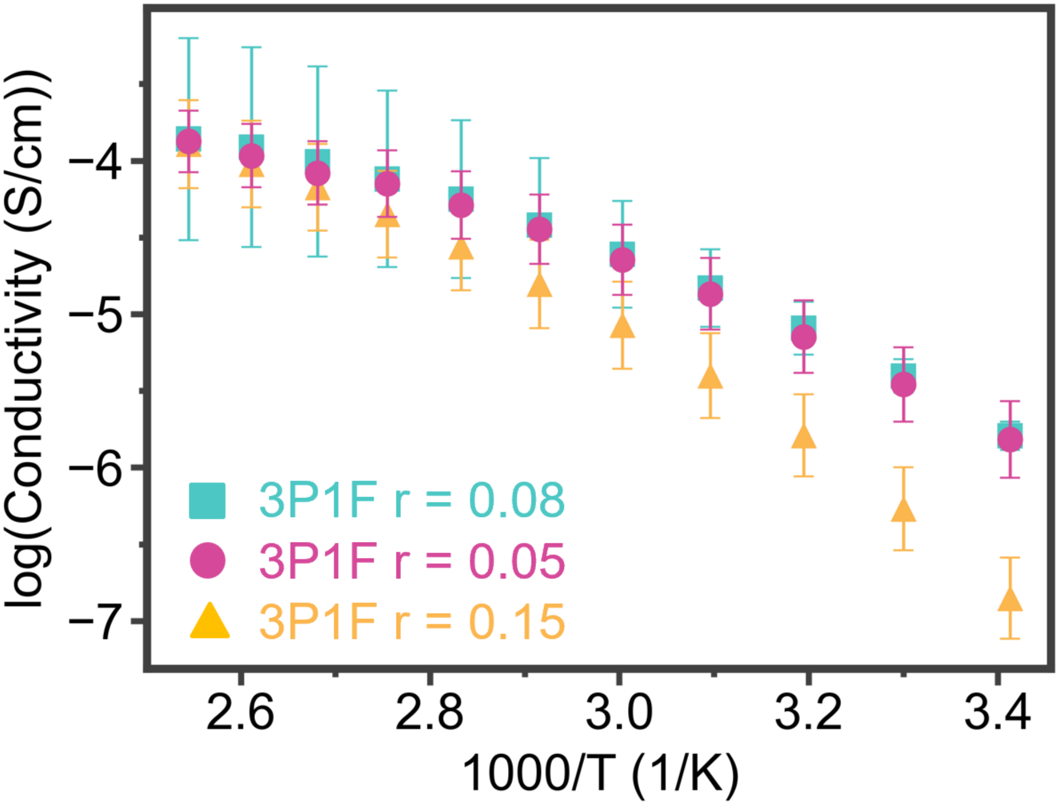

Figure S3. Dependence of ionic conductivity on salt content in the 3P1F polymer blend electrolyte

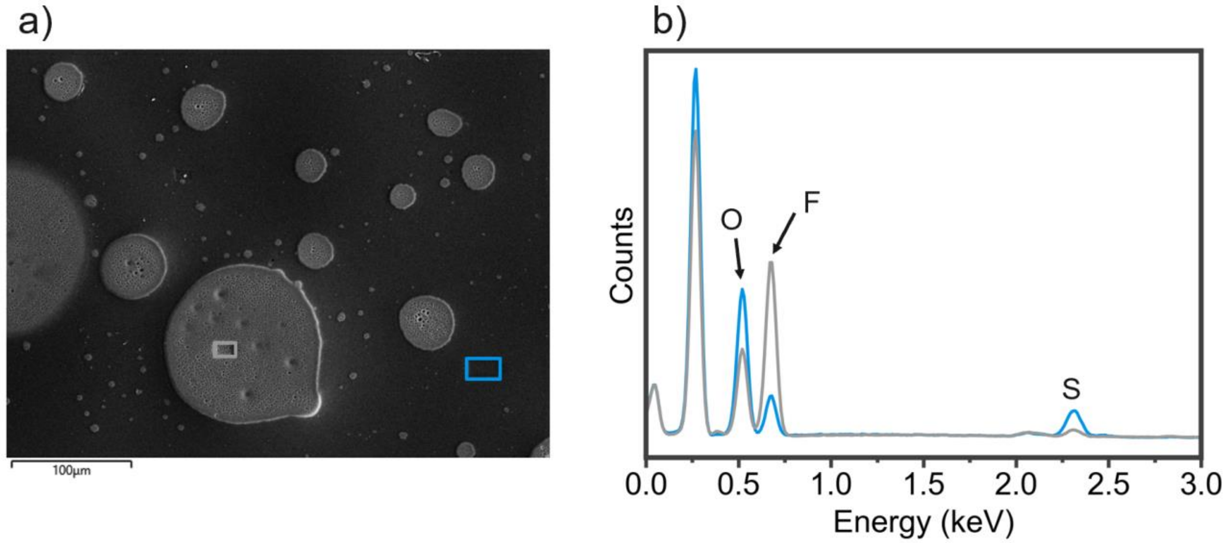

Figure S4. a) SEM image of the area investigated with EDX, where the gray rectangle and blue rectangle were the two spots chosen. **b)** The spectra from the two spots shows higher fluorine content and lower oxygen content in the gray phase, which identifies this phase as PFPE. The spectra also show a higher content of sulfur ($TFSI^-$) in the PEG phase.

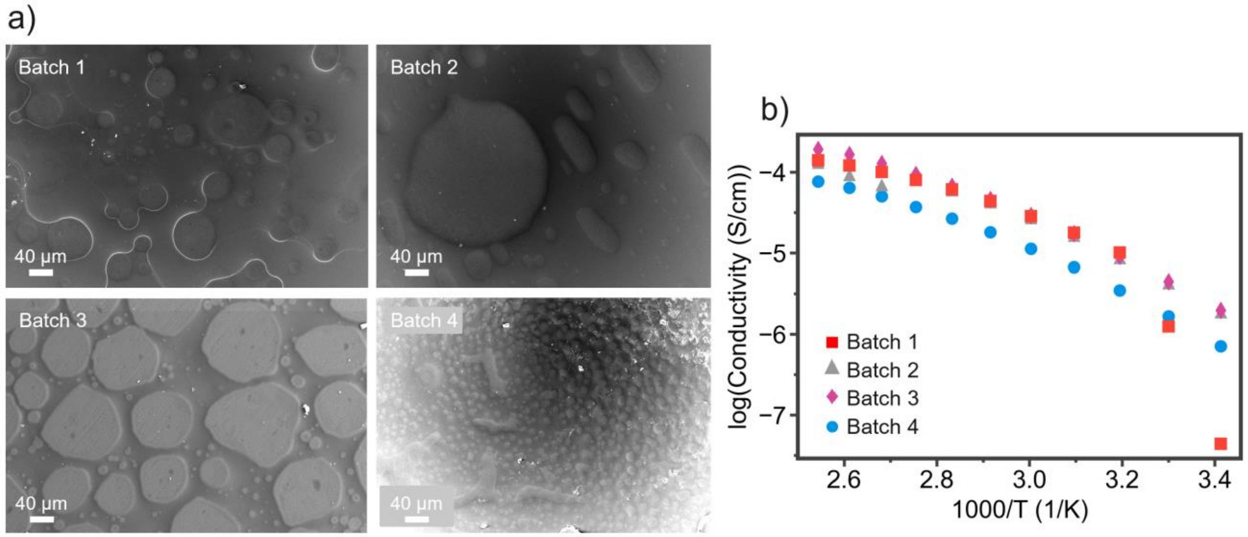

Figure S5. a) SEM images for 4 different batches of 3P1F r = 0.05 LiTFSI films, which show very similar performance as outlined in **b)** the conductivity data for the 4 batches.

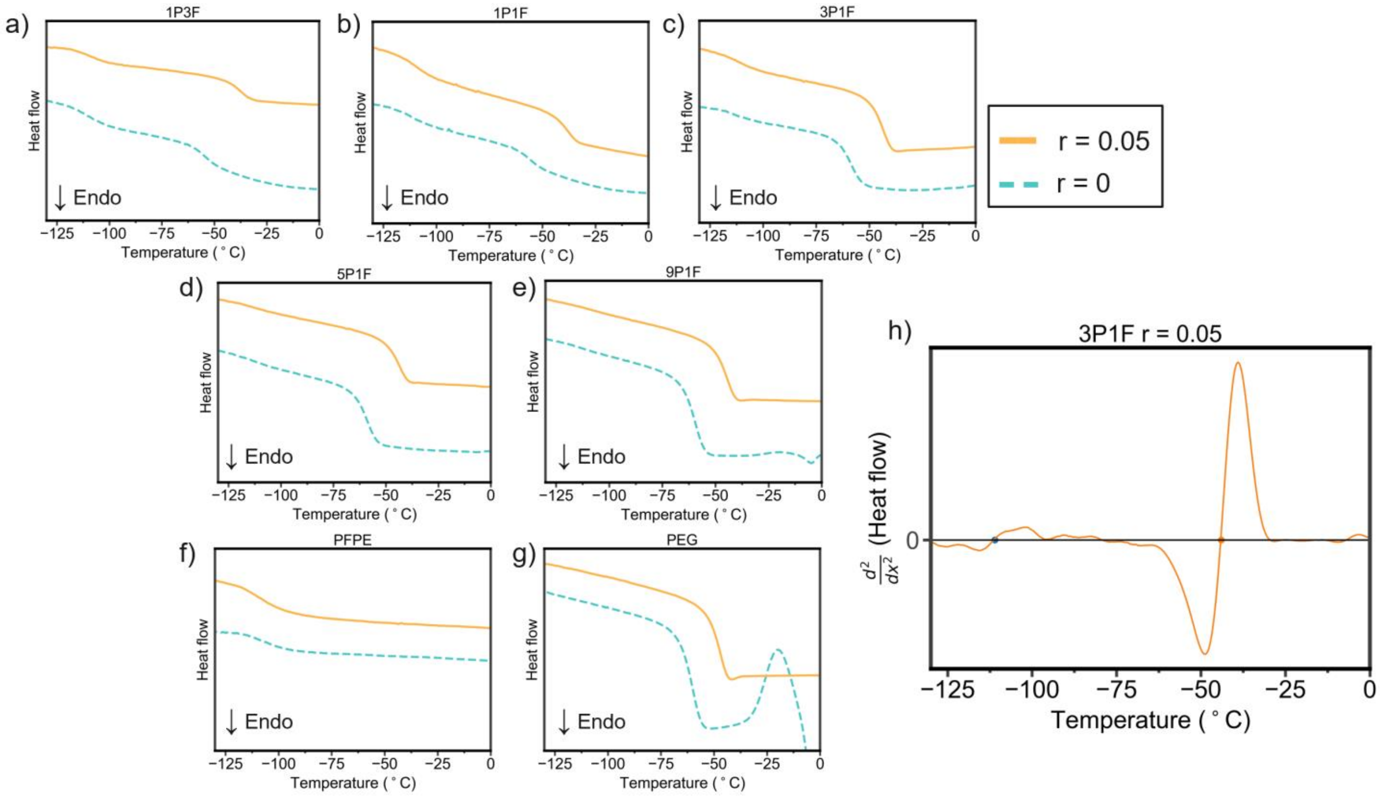

Figure S6. a) – g) DSC heating curve for 1P3F, 1P1F, 3P1F, 5P1F, 9P1F, PFPE, and PEG samples, respectively, showing both the samples with salt and samples without salt. **h)** Derivative method used to find the T_g of the PFPE and PEG phases, where the T_g is the temperature where the second derivative of the heat flow curve equals zero.

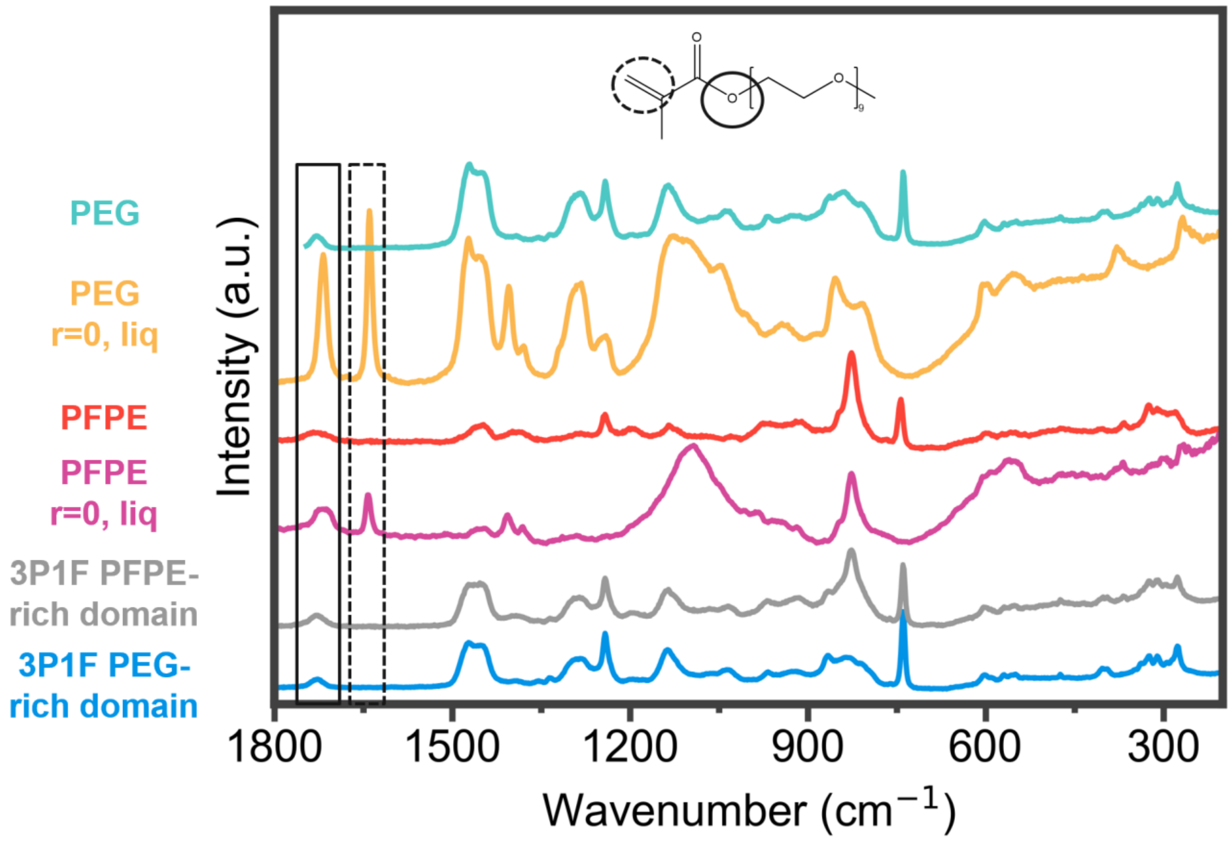

Figure S7. Raman data normalized to the ester peak at ~1740 cm^{-1}. This normalization allows for direct comparison of the salt peak intensities between phases. Disappearance of the vinyl peak in the crosslinked samples shows full crosslinking of all end groups. All samples include salt with r = 0.05 except the liquid PEG and PFPE, which have no salt.

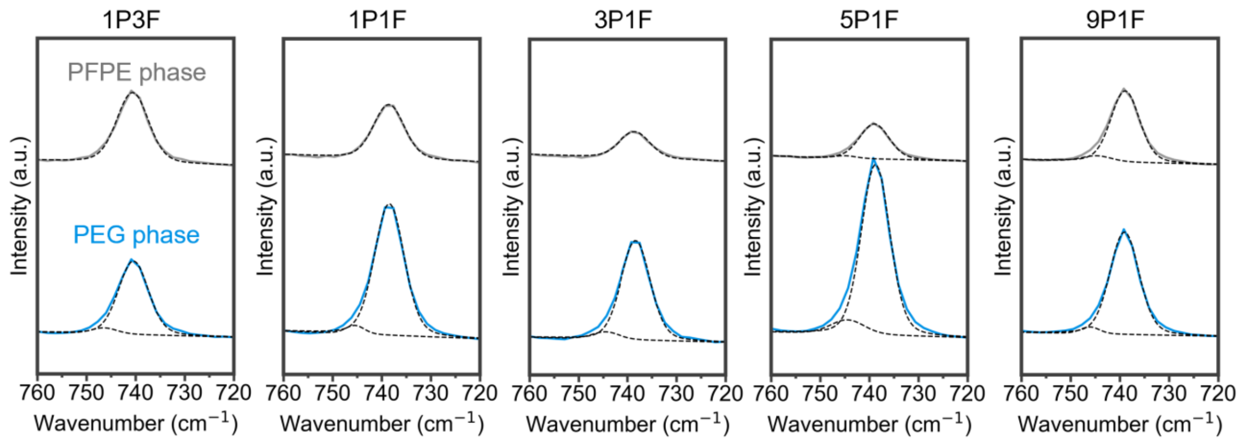

Figure S8. Raman salt dissociation in the PFPE phase (gray) and the PEG phase (blue) for 1P3F, 1P1F, 3P1F, 5P1F, and 9P1F.

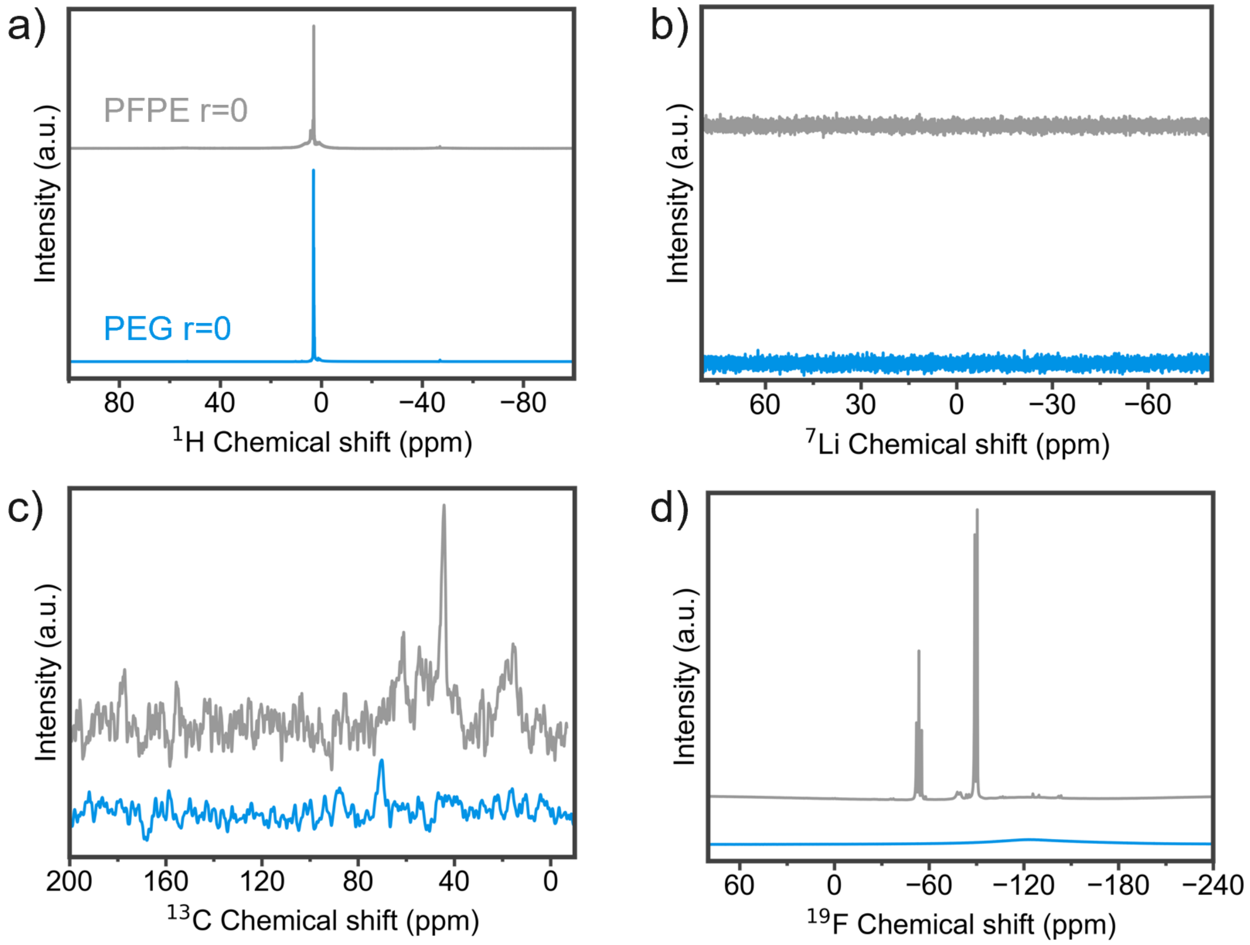

Figure S9. a) ^{1}H, **b)** ^{7}Li, **c)** ^{13}C, and **d)** ^{19}F MAS NMR spectra for PFPE and PEG r = 0 controls

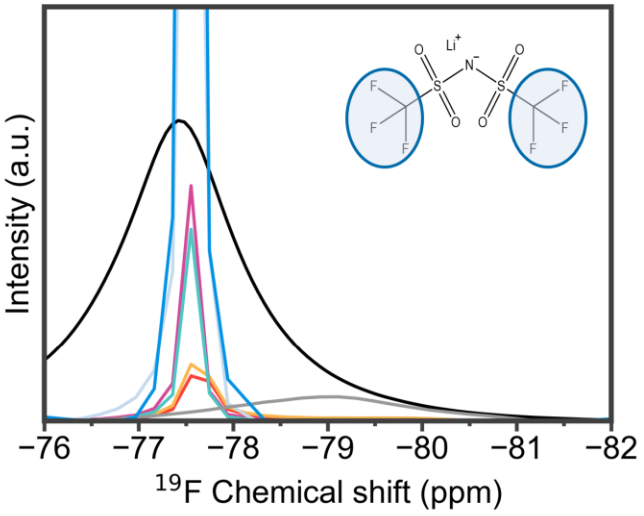

Sample	Chemical shift (ppm)	Linewidth (Hz)
PFPE r = 0.05	-78.94	821.59
1P3F r = 0.05	-77.45	163.19
1P1F r = 0.05	-77.45	164.36
3P1F r = 0.05	-77.45	70.09
5P1F r = 0.05	-77.45	68.63
9P1F r = 0.05	-77.45	69.90
PEG500 r = 0.05	-77.45	64.51
LiTFSI	-77.40	527.91

Figure S10. ^{19}F MAS NMR of the $TFSI^{-}$ peak for all polymer blends and controls, along with linewidth analysis.

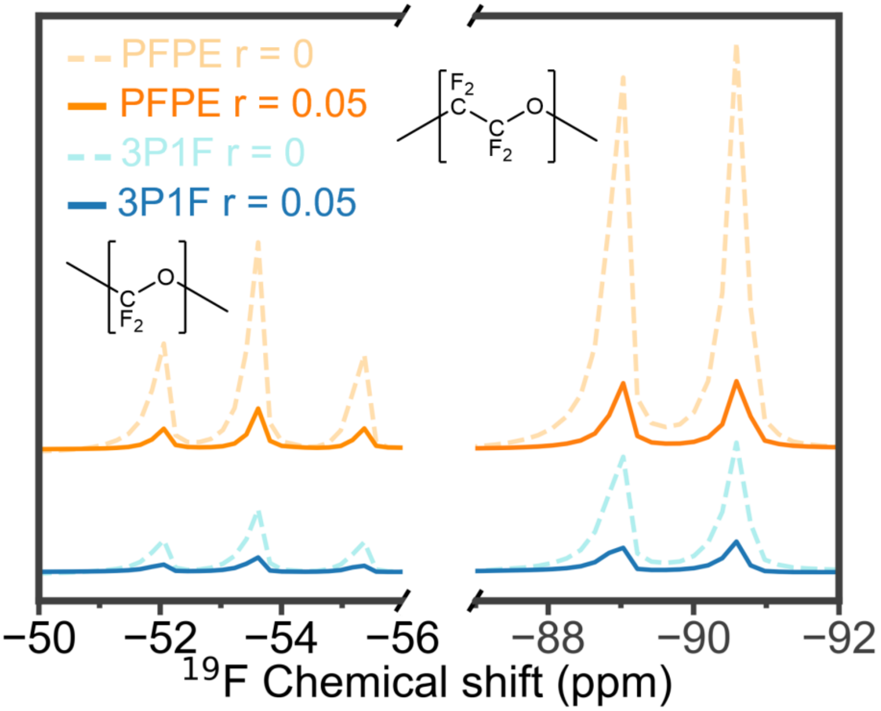

Figure S11. ^{19}F MAS NMR for r = 0 and r = 0.05 PFPE and 3P1F films in the domains showing the $-CF_2-O-$ and $-CF_2-CF_2-O-$ repeat units[3]. No shifting occurs in the CF_2 backbone peaks between the pristine and the r = 0.05 samples, showing that there is no observable PFPE-$TFSI^-$ interaction.

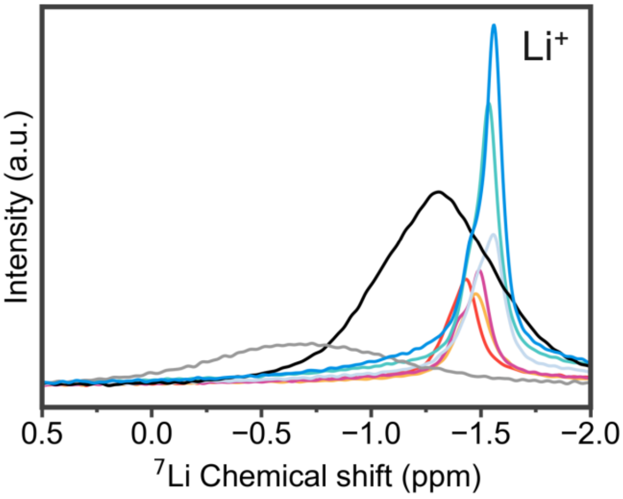

Sample	Chemical shift (ppm)	Linewidth (Hz)
PFPE r = 0.05	-0.70	155.19
1P3F r = 0.05	-1.43	27.05
1P1F r = 0.05	-1.48	28.12
3P1F r = 0.05	-1.49	26.59
5P1F r = 0.05	-1.54	21.76
9P1F r = 0.05	-1.55	28.60
PEG500 r = 0.05	-1.56	18.03
LiTFSI	-1.312	95.55

Figure S12. ^{7}Li MAS NMR of the Li^+ peak for all polymer blends and controls, along with linewidth analysis.

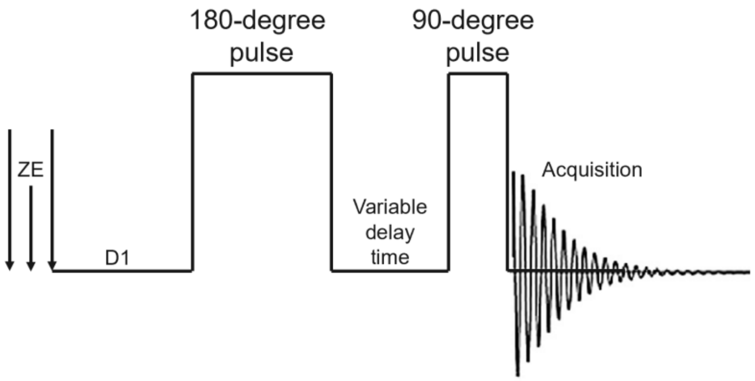

Figure S13. T_1 Experiment 't1ir' Pulse Sequence

<u>T_1 Experiment 't1ir' Details:</u>

The T_1 experiment uses a two-pulse program with a variable delay time between the two pulses. First, the program applies a 180-degree pulse to invert the orientation of the nuclei. The nuclei then relax back towards their original orientations in the B_0 field over the variable delay time, after which a 90-degree pulse is applied, immediately followed by detection. The program is run a total of 12 times, with 12 different delay times. As the delay time increases, the intensity of the Li^+ peak increases until it reaches a maximum plateau value. This characterizes the speed of the relaxation of the target nuclei, where all of the nuclei will have relaxed after the characteristic time is reached, and the 90-degree pulse will project the nuclei into the x,y field where they are detected together creating the maximum intensity. When lower delay times are used, fewer nuclei have fully relaxed, and the measured intensity is lower. The data for the peak intensity with variable delay time is fit with an exponential function given in Equation S1 to determine the characteristic spin-lattice relaxation time, T_1.

$$I = Ae^{t/T_1} \qquad \text{Equation S1}$$

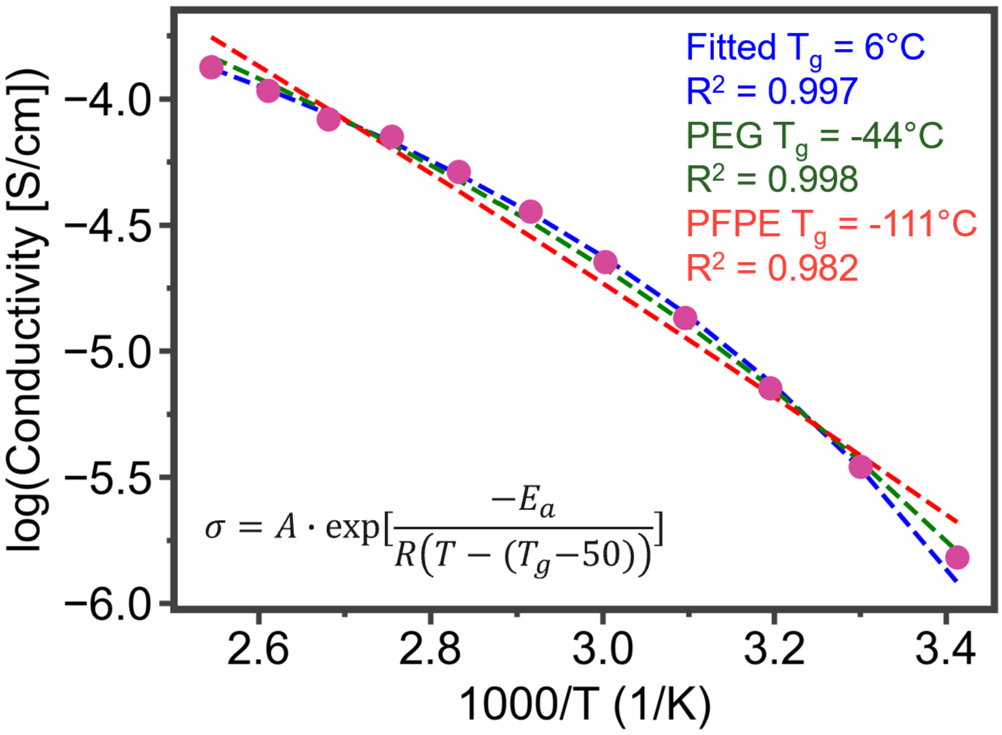

Figure S14. VTF fittings via different fitting techniques for the 3P1F r = 0.05 LiTFSI polymer. First, the curve_fit function from the SciPy library in Python was permitted to fit the T_g value, then the PEG phase T_g was set in the program and finally the PFPE phase T_g was set in the program. The PEG phase T_g yielded the best fitting. R^2 values were calculated using the r2_score function from the Scikit-learn library in Python.

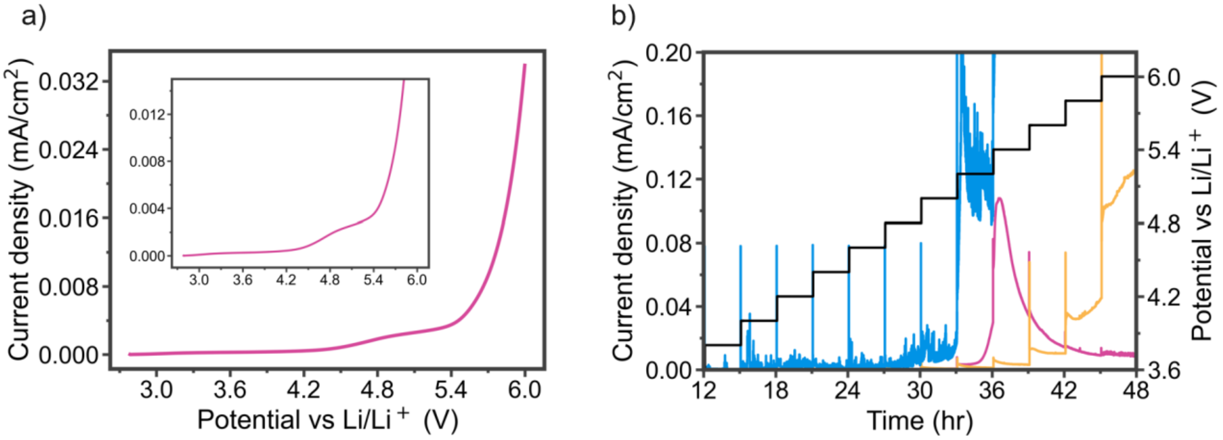

Figure S15. Li|SS Oxidative Stability Testing **a)** Linear sweep voltammetry and **b)** potentiostatic hold experiments

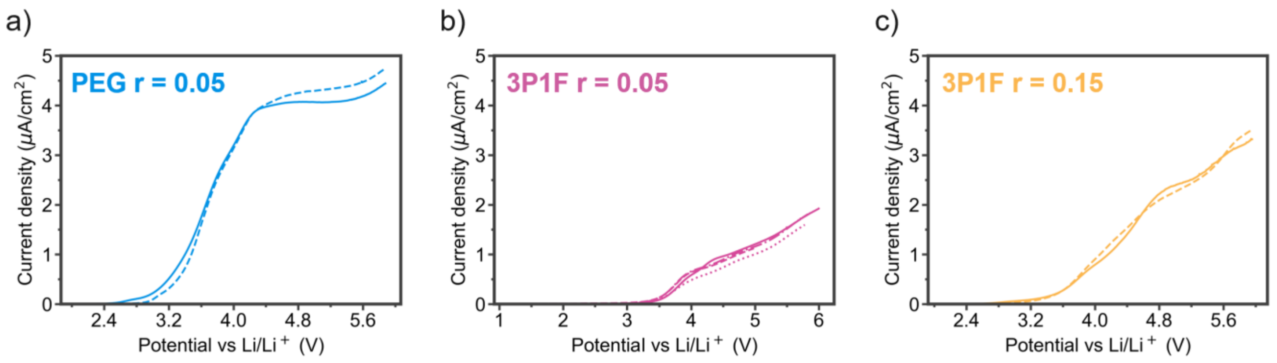

Figure S16. Linear Sweep Voltammetry replicates for **a)** PEG r = 0.05, **b)** 3P1F r = 0.05, and **c)** 3P1F r = 0.15 in Li|Al cells. Each plot shows the voltammogram plotted in the main manuscript along with the replicate(s). **b)** shows 4 total voltammograms.

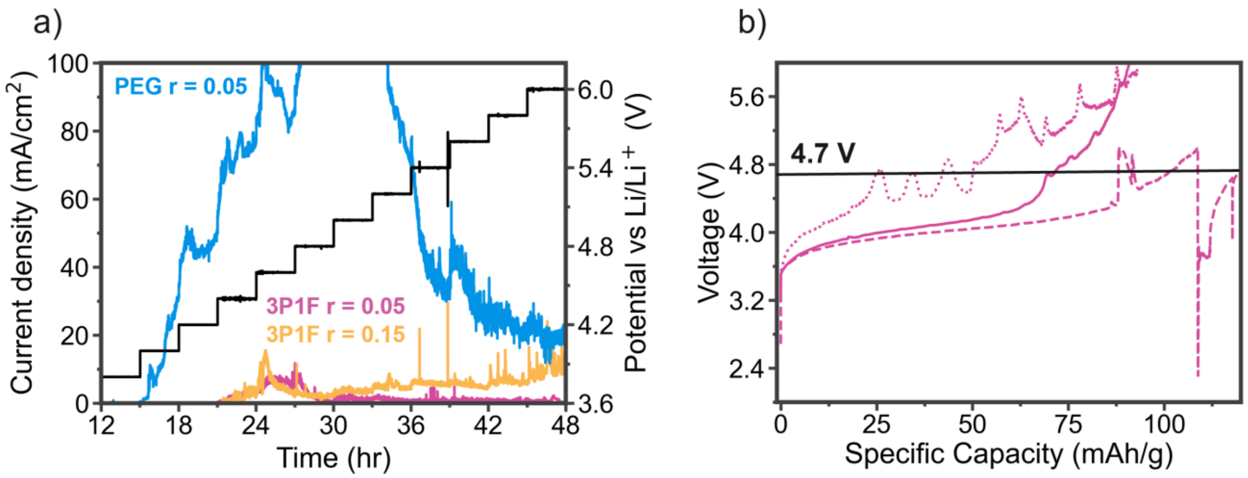

Figure S17. Oxidative stability testing with an LFP ($LiFePO_4$) working electrode **a)** Potentiostatic hold experiments for Li|LFP cells with the 3P1F r = 0.05, 3P1F r = 0.15, and PEG r = 0.05 polymer electrolytes, where each potential was held for 3 hours. Oxidative current from 3.6 – 4.4 V is likely due to delithiation reactions, and the baseline current after 42 hours is likely tied to continual oxidation of the electrolyte. **b)** Galvanostatic charge of three Li|3P1F r = 0.05 LiTFSI|LFP coin cell samples showing delithiation between 3.6 – 4.4 V. Starting at 4.7 V, after the delithiation stage, electrolyte degradation is observable in the voltage noise and continual increase in specific capacity.

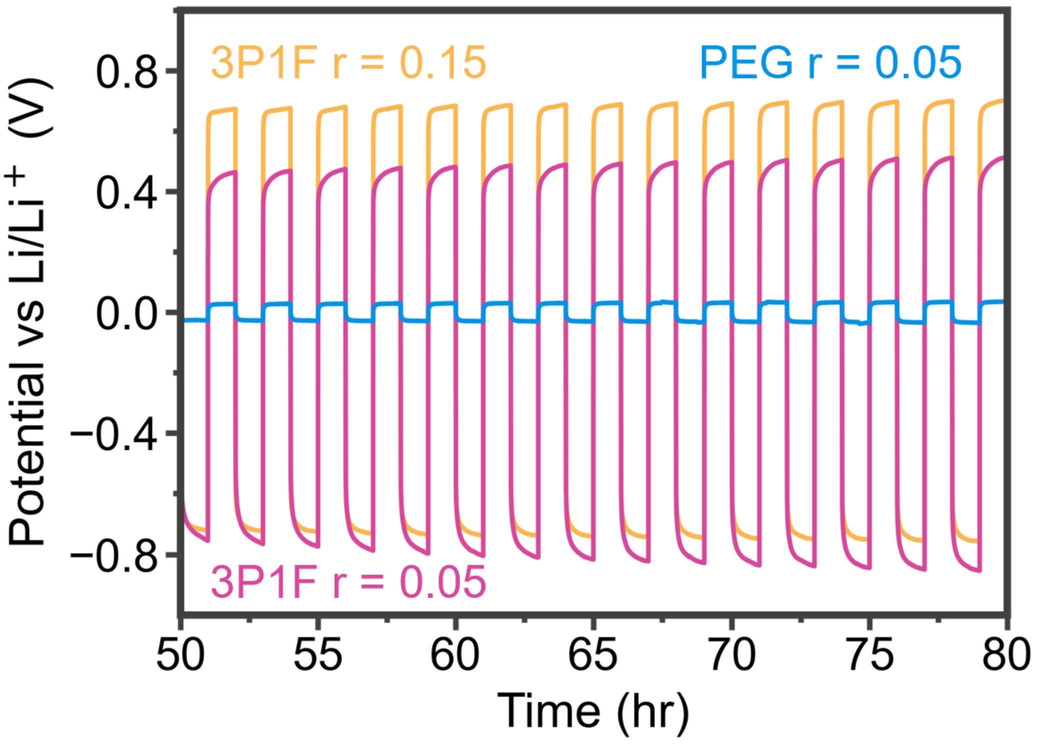

Figure S18. Enlarged Li|Li cycling profiles for 3P1F r = 0.05, 3P1F r = 0.15, and PEG r = 0.05 cycled at 0.1 mA/cm^2.

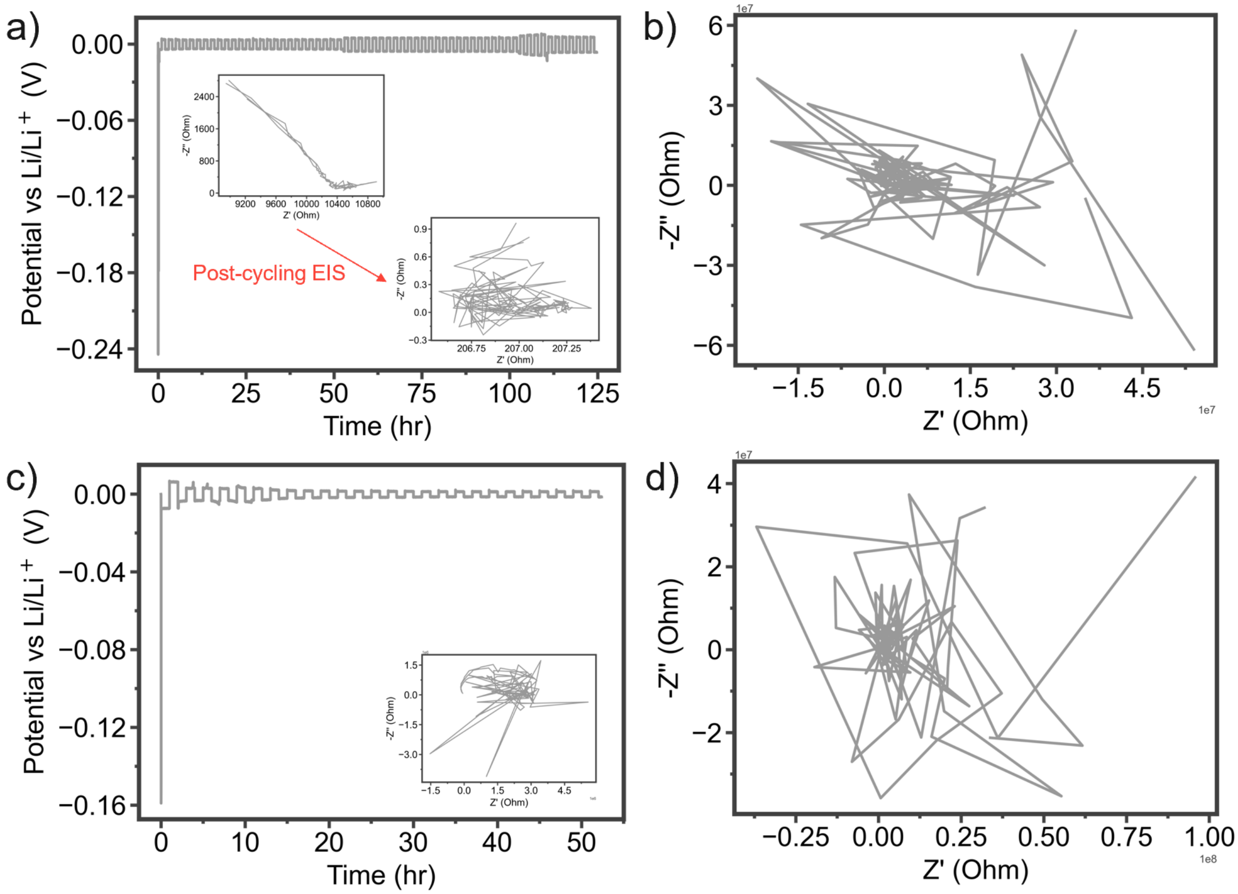

Figure S19. Li|Li cycling attempts for PFPE r=0.05 film. **a)** and **c)** show voltage profiles for two cells that short circuited immediately after the cycling experiment began. Insets in these images show the EIS spectra pre- (only **a**) and post-cycling (**a** and **c**). Most PFPE cells that were fabricated showed poor contact between the rigid film and the lithium metal, as shown in the EIS spectra in **b)**, **c)**, and **d)**, which impeded Li plating and stripping at the polymer-electrode interface.

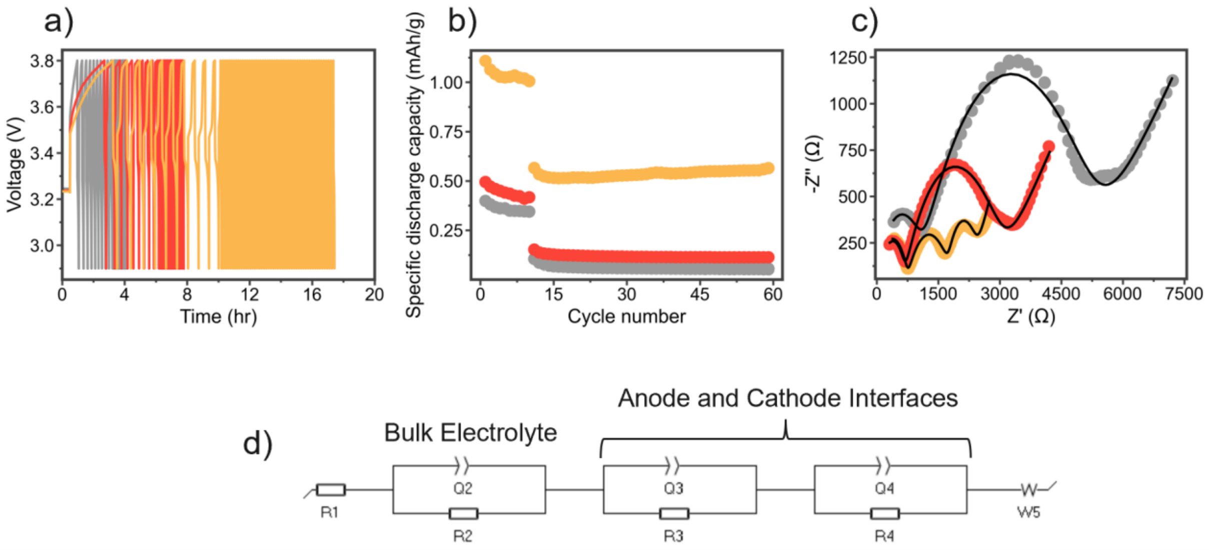

Figure S20. Li|LFP cycling with 3P1F r = 0.05 LiTFSI with the **a)** potential profile and **b)** specific discharge capacity for three coin cell replicates. **c)** Post cycling EIS shows the comparison of charge transfer resistance at the solid|solid interface and bulk electrolyte resistance between each of the cells. **d)** Equivalent circuit generated in EC-Lab® software (BioLogic) for the Li|LFP cell with solid polymer electrolyte.[4]

Li|LFP cycling

As seen in the Li|Li cycling in **Figure 6**, the overpotential to Li plating and stripping is very high, which translates to high overpotential to Li^+ (de)intercalation at the LFP interface and poor cycling (**Figure S20**). **Figure S20a** shows the voltage profile associated with Li|LFP cell cycling at C/40 for 10 cycles and C/20 for 50 cycles at 80°C. This cycling protocol was performed after a 24-hour rest period at 80°C to accommodate electrode|electrolyte interface formation. The specific discharge capacity, shown in **Figure S20b**, is less than 1% of LFP's theoretical capacity; however, we can see at C/40 that some degree of intercalation or other electrochemical reaction occurs at the electrode interface before the reactions become too impeded and the high overpotential to electrochemical reactions push the potential to the cut off value. In the C/20 cycles this does not happen, and the current is too fast, immediately reaching the cut off potential. It is important to note that increasing the cutoff potential to 5.5V as shown in Figure S17b does make it possible to extract even larger capacities from LFP.

There is some variability between the three cells, and the post-cycling EIS in **Figure S20c** shows that this is likely due to the wide range of resistance stemming from degradation layer growth at the anode and cathode interfaces. These two features are represented in the second and third low frequency features in the Nyquist plot, as diagramed in the equivalent circuit in **Figure S20d**.[4] The best performing cell (yellow data) shows three small, well resolved Nyquist plot features that have a low total resistance associated with them. This correlates well with the observation that this cell reaches the highest discharge capacities during cycling.

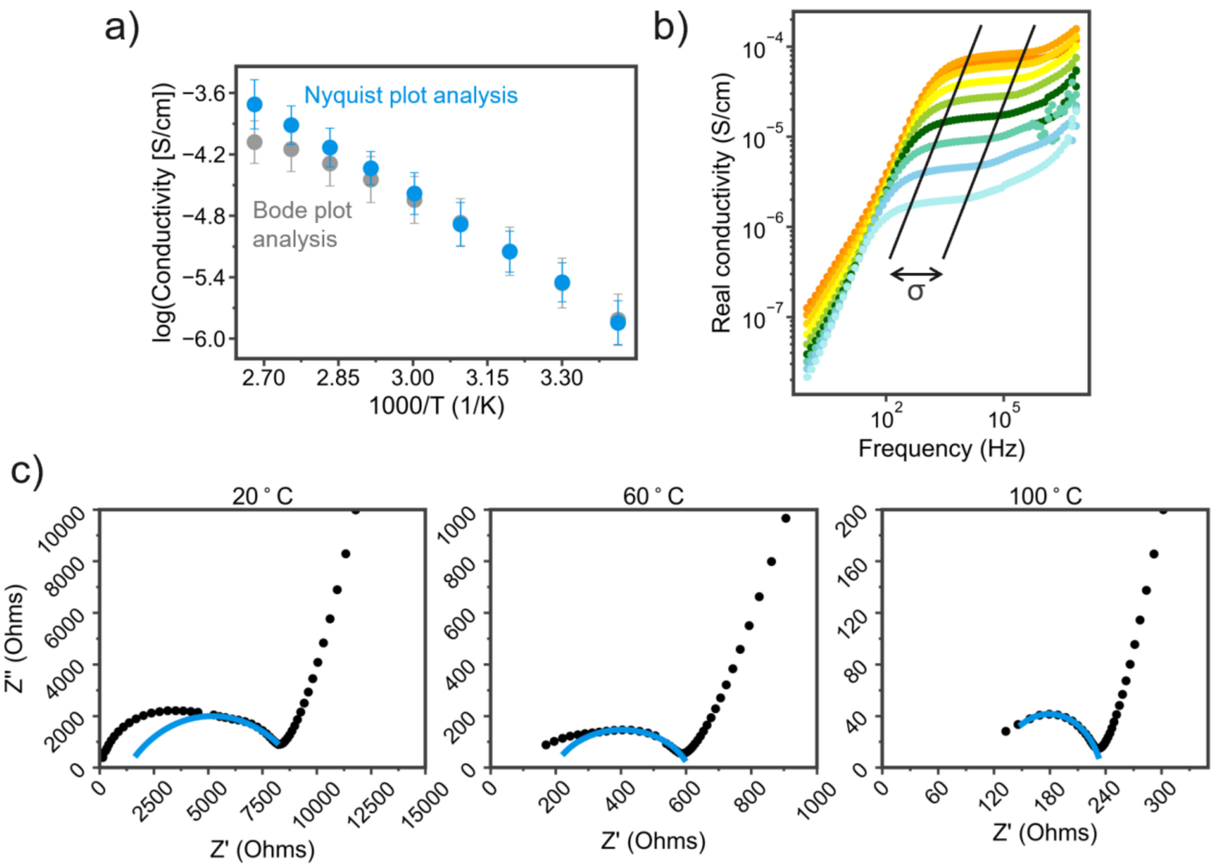

Figure S21. Conductivity analysis of the 3P1F r = 0.05 LiTFSI polymer. **a)** Conductivities calculated from the Nyquist and Bode plot methods for three coin cells. **b)** Bode plot showing the frequency dependent conductivity and the plateau region that was used to calculate the conductivity at each temperature. **c)** Nyquist impedance plots at 20, 60, and 100°C with curves fitted to the feature indicative of the ionic conductivity using the Randles circuit.

The conductivity was determined via two methods, analysis of the frequency dependent conductivity data in the Bode plot and analysis of the equivalent circuit fitting of the Nyquist plot. As shown in **Figure S21a**, the Bode plot and Nyquist plot analysis yield the same results at low temperature but show diverging behavior at higher temperature. The Bode plot analysis[5,6] is used to calculate the conductivity of the samples in this work, since the Nyquist plot shows convolution of the high frequency and mid-range frequency circuit features that add more error to the conductivity calculation.

<u>Bode Plot Analysis:</u>

The loss factor (imaginary component of the complex permittivity) can be calculated from the impedance data via Equation S2:

$$\epsilon'' = \frac{Z\prime}{\omega c_0 (Z'^2 + Z''^2)} \qquad \text{Equation S2}$$

The angular frequency and c_0 are defined by Equations S3 and S4, where f is the frequency in Hz, ϵ_0 is vacuum permittivity, A is the electrochemically active surface area, and t is the thickness of the electrolyte:

$$\omega = 2\pi f \text{ and } c_0 = \frac{\epsilon_0 A}{t}$$ Equations S3 and S4

The loss factor is used to calculate the frequency dependent conductivity via Equation S5:

$$\sigma(\omega) = \epsilon'' \omega \epsilon_0$$ Equation S5

The conductivity is plotted in **Figure S21b** for spectra obtained at 9 temperatures from 100-20℃. The ionic conductivity for the sample at a given temperature was calculated by finding the average conductivity value along the plateau in the $\sigma(\omega)$ spectra.

Nyquist Plot Analysis:

The Nyquist plot was fitted with the Randles circuit to R_1 and R_2 values. This circuit was fitted specifically to the second feature in the plot as shown in **Figure S21c**. As the temperature decreased, the presence of a high frequency feature became prominent which is likely due to interfacial resistances that are more prevalent at low temperatures. R_2 was used to calculate the conductivity from the Nyquist plot with Equation S6.

$$\sigma = \frac{t}{R_2 A}$$ Equation S6

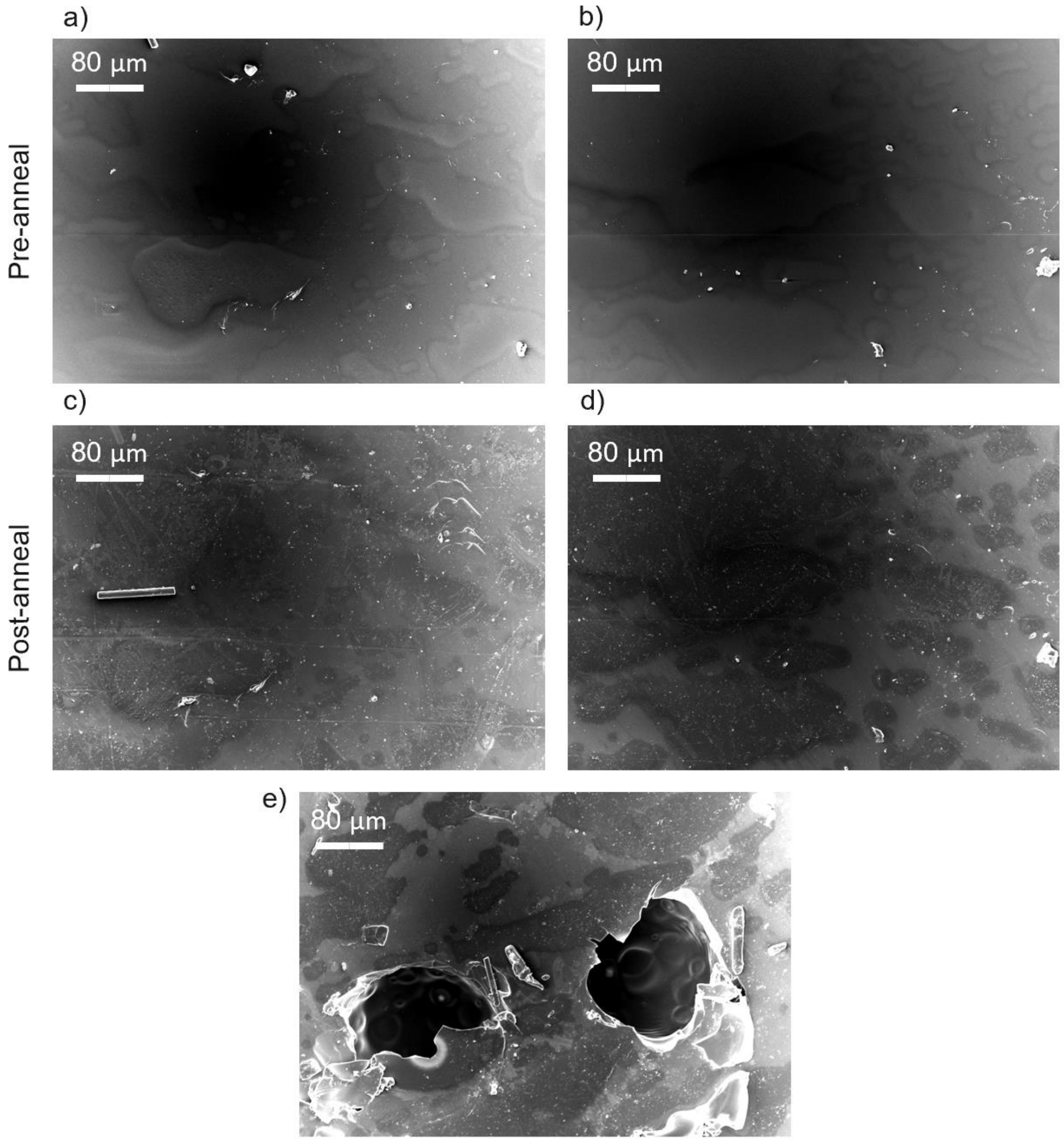

Figure S22. Annealing test of 3P1F r = 0.05 LiTFSI film at 80°C showing preservation of polymer surface morphology with long term heating in coin cells. **a)** and **b)** show spots 1 and 2, respectively, of the polymer film pre-annealing, while spots **c)** and **d)** show spots 1 and 2, respectively, post-annealing. **e)** Two tears resulting from the polymer extraction method show that the PFPE and PEG phases both appear to continue throughout the polymer bulk, and neither exists solely at the polymer interface.

<u>3P1F Annealing Test:</u>

The 3P1F r = 0.05 LiTFSI film was first mounted onto a zinc disk and prepared for SEM imaging. After imaging, the zinc disk and film were loaded into a coin cell in an argon filled glovebox with an opposing stainless steel disk to form a Zn|3P1F r = 0.05 LiTFSI|SS cell. This cell was crimped and put into an electrochemical testing coin cell clip in an 80°C oven for 12 hours. This coin cell preparation method matches the method used to prepare coin cells for electrochemical testing in the manuscript. After the 12-hour annealing period at 80°C, we decrimped the coin cell in an argon filled glovebox and extracted the

Zn disk with the polymer still attached. Some tearing and abrasion occurred during the extraction process. This sample was then imaged with SEM.

Two spots imaged during the pre-annealing SEM session were found and re-imaged after the annealing process. Spot 1 is shown in **Figure S22a** pre-annealing and **Figure S22c** post-annealing, and spot 2 is shown pre-annealing in **b)** and post-annealing in **d)**. For both spots, the pre-annealing and post-annealing morphologies match, showing no difference in PEG-PFPE phase morphology with heating. This is due to the physical stabilization induced by the tough PFPE phases. Along with the pre- and post-annealing images, we have included a post-annealing image of a tear formed during the extraction process, **Figure S22e**. Inside the hole you can see small particulates jutting out of the matrix that are likely PFPE phases within the PEG matrix. This glimpse inside the film shows that the PEG and PFPE phases both exist throughout the bulk of the film, not just at the interface. This shows that morphology changes due to high temperature annealing are not the cause of enhanced oxidative stability in the linear sweep voltammetry and potentiostatic hold tests.

Supplementary Table

Table S1. Oxidative stability calculations with literature cross-validation

Compound	V_{ox} (V vs Li/Li^+), this work	V_{ox} (V vs Li/Li^+), literature	HOMO (eV), this work	HOMO (eV), literature
**m-PEG**	5.29	5.1,[7] 6.13[8]	-7.01	-6.98[9]
**m-PFPE**	7.57	6.88[7]*	-9.39	-8.72[7]*
**$TFSI^-$**	5.93	5.68,[10] 5.71,[11] 6.02[11]	-8.14	-8.44[10]

*PFPE calculations in the investigation carried out by Pandian et al. use a PFPE chain with diol end groups. The HOMO position is on the alcohol groups, which are more susceptible to oxidation than the methyl end groups on our m-PFPE chains. This contributes to the discrepancy between our calculations.

References

(1) Devaux, D.; Bouchet, R.; Glé, D.; Denoyel, R. Mechanism of Ion Transport in PEO/LiTFSI Complexes: Effect of Temperature, Molecular Weight and End Groups. *Solid State Ionics* **2012**, *227*, 119–127.

(2) Rohatgi, A. WebPlotDigitizer. Pacifica, California, USA 2022.

(3) Amanchukwu, C. V; Gunnarsd, A. B.; Choudhury, S.; Newlove, T. L.; Magusin, P. C. M. M.; Bao, Z.; Grey, C. P. Understanding Lithium-Ion Dynamics in Single-Ion and Salt-in- Polymer Perfluoropolyethers and Polyethyleneglycol Electrolytes Using Solid-State NMR. *Macromolecules* **2023**, *56*, 3650–3659.

(4) Vadhva, P.; Hu, J.; Johnson, M.; Stocker, R.; Braglia, M.; Brett, D.; Rettie, A. Electrochemical Impedance Spectroscopy for All-Solid-State Batteries Theory Methods and Outlook. *ChemElectroChem* **2021**, *8*, 1930–1947.

(5) Lvovich, V. F. *Impedance Spectroscopy: Applications to Electrochemical and Dielectric Phenomena*; Wiley: Hoboken, NJ, 2012.

(6) Popov, I.; Cheng, S.; Sokolov, A. P. Broadband Dielectric Spectroscopy and Its Application in Polymeric Materials. In *Macromolecular Engineering: From Precise Synthesis to Macroscopic Materials and Applications*; Matyjaszewski, K., Gnanou, Y., Hadjichristidis, N., Muthukumar, M., Eds.; Wiley-VCH Gmbh, 2022; pp 1–39.

(7) Pandian, S.; Adiga, S. P.; Tagade, P.; Hariharan, K. S.; Mayya, K. S.; Lee, Y. G. Electrochemical Stability of Ether Based Salt-in-Polymer Based Electrolytes: Computational Investigation of the Effect of Substitution and the Type of Salt. *J. Power Sources* **2018**, *393*, 204–210.

(8) Marchiori, C. F. N.; Carvalho, R. P.; Ebadi, M.; Brandell, D.; Araujo, C. M. Understanding the Electrochemical Stability Window of Polymer Electrolytes in Solid-State Batteries from Atomic-Scale Modeling: The Role of Li-Ion Salts. *Chem. Mater.* **2020**, *32* (17), 7237–7246.

(9) Chen, L.; Venkatram, S.; Kim, C.; Batra, R.; Chandrasekaran, A.; Ramprasad, R. Electrochemical Stability Window of Polymeric Electrolytes. *Chem. Mater.* **2019**, *31* (12), 4598–4604.

(10) Han, J.; Balbuena, P. B. First-Principles Calculations of Oxidation Potentials of Electrolytes in Lithium – Sulfur Batteries and Their Variations with Changes in Environment. *Phys. Chem. Chem. Phys.* **2018**, *20*, 18811–18827.

(11) Borodin, O.; Behl, W.; Jow, T. R. Oxidative Stability and Initial Decomposition Reactions of Carbonate , Sulfone , and Alkyl Phosphate-Based Electrolytes. *J. Phys. Chem. C* **2013**, *117*, 8661–8682.
